# UPLC-MS/MS-GNPS based metabolite profiling and inhibitory potential of *Vitex negundo* L. leaves against Shiga toxins: Applications for biodegradable films with antibacterial properties

**DOI:** 10.1016/j.btre.2026.e00987

**Published:** 2026-10-19

**Authors:** Nguyen-Huan Pham-Khanh, Thi-Kim-Quy Ha, Do-Thanh-Phong Truong

**Affiliations:** College of Natural Sciences, Can Tho University, Campus II, 3/2 street, Ninh Kieu ward, Can Tho City, 94000, Vietnam

**Keywords:** *Vitex negundo*, Shiga toxin, *Escherichia coli* O157:H7, Feature-based molecular networking, Biodegradable films

## Abstract

Antibiotic therapy for STEC O157:H7 infections induces Shiga toxin release, highlighting the need for alternative, food-compatible antimicrobial strategies. The ethanolic leaf extract of *Vitex negundo* L. was characterized by UPLC-qTOF-MS/MS, integrated with Feature-Based Molecular Networking (GNPS), which putatively annotated 56 metabolites. Computational screening of all annotated compounds against the Stx1 and Stx2 B-subunits identified compound 8, an iridoid glycoside, as the top-ranked candidate with predicted Stx1-binding potential (ΔG = -6.9 kcal/mol; K*i* = 8.7 µM). Over 100 ns of molecular dynamics, the Stx1 B-subunit backbone remained conformationally stable (Cα RMSD < 1.5 Å) and 2–3 intermolecular hydrogen bonds with compound 8 were maintained; ADMET prediction returned a gut-restricted profile. A sweet potato starch-based film incorporating the crude extract showed antibacterial activity against *E. coli* O157:H7 and complete visual disintegration within 28 days of soil burial, indicating preliminary potential as an eco-friendly active packaging material for dried foods.

## Highlights

• GNPS molecular networking mapped 56 metabolites in *V. negundo* leaves.
• Compound 8 matched the reference Stx1 inhibitor in docking (ΔG = −6.9 kcal/mol).
• Over 100 ns MD, the Stx1 backbone stayed stable with 2 − 3 H-bonds to compound 8.
• Iridoid (compound 8) is predicted to act as a gut-restricted anti-Shiga toxin lead.
• *V. negundo*/starch films inhibited STEC and visually disintegrated within 28 days.

## Graphical abstract

**Figure:**
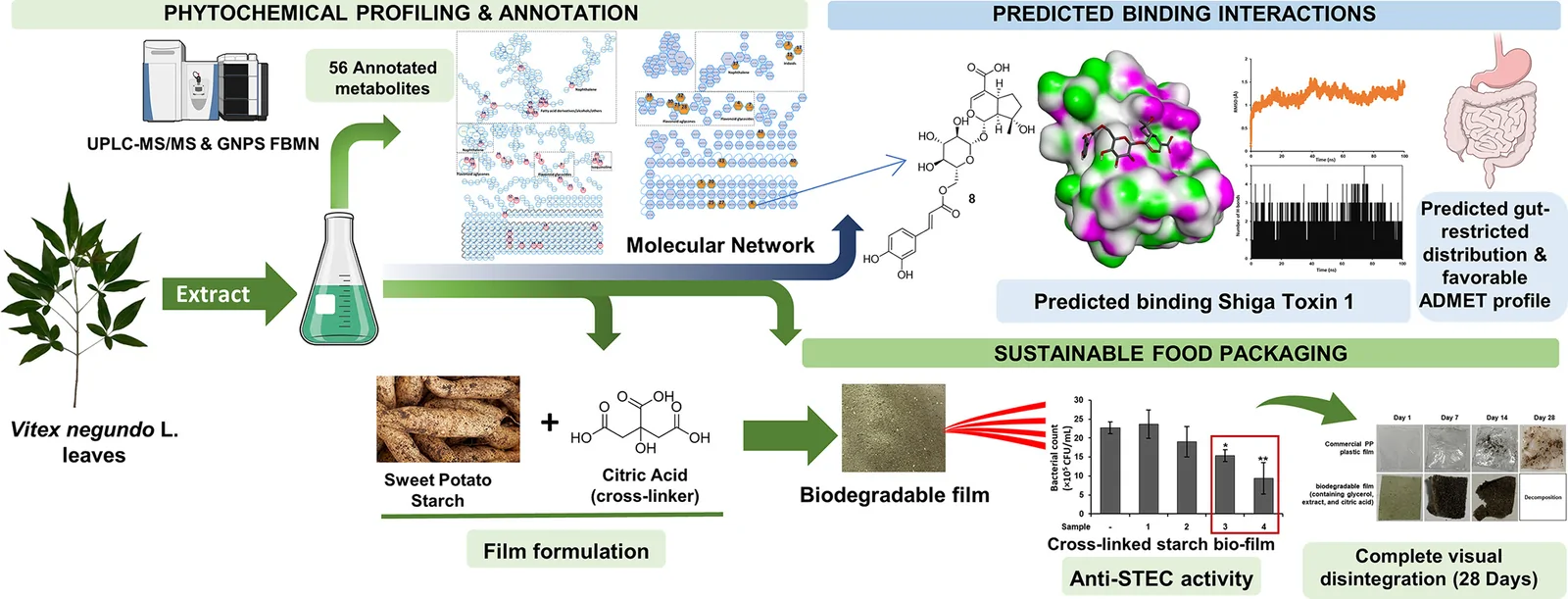

## 1 Introduction

Shiga toxin-producing *E. coli* (STEC) O157:H7 is among the most significant foodborne pathogens threatening global food safety [1,2]. The pathogenicity of STEC O157:H7 is primarily attributed to two Shiga toxins (Stx1 and Stx2), which irreversibly inhibit protein synthesis by depurinating the 28S rRNA of host ribosomes, leading to severe clinical outcomes including hemorrhagic colitis and hemolytic uremic syndrome (HUS) in 5–15% of infected individuals [2,3]. STEC causes >1 million illnesses worldwide annually [2]. Control of STEC O157:H7 in food systems is further complicated by the contraindication of conventional antibiotics, which trigger the bacterial SOS response and amplify Shiga toxin release [2,4], necessitating the development of alternative, food-compatible antimicrobial strategies.

The significant clinical and economic impact of STEC O157:H7 infections requires a comprehensive, dual-approach prevention strategy: preventing bacterial transmission on food surfaces and neutralizing Shiga toxins following ingestion. From a food safety standpoint, developing biodegradable packaging materials that incorporate crude plant extracts is a promising preventive strategy [5]. This strategy provides an initial barrier to STEC colonization at the packaging level while addressing environmental concerns about plastic waste accumulation [6]. When foodborne transmission occurs despite preventive measures, conventional antibiotic therapy is contraindicated [7]. This therapeutic limitation highlights the critical need for an alternative. Therefore, characterizing bioactive compounds in plant extracts to identify molecules capable of neutralizing Shiga toxins is a valuable research direction for therapeutic development [8]. Such compounds should ideally demonstrate gut-restricted activity to minimize systemic effects while maintaining efficacy against the target toxins. This integrated approach, combining preventive packaging technologies with targeted antitoxin therapies, offers a comprehensive framework for addressing STEC O157:H7-related public health challenges.

Plant-derived secondary metabolites − including polyphenols, flavonoids, terpenoids, and essential oils − have emerged as promising candidates for natural food preservation and safety strategies against STEC O157:H7 [9,10]. Among the plant species with documented antimicrobial potential, *Vitex negundo* L. is a medicinal shrub native to tropical and subtropical Asia and parts of Eastern Africa [11,12], with a well-established history in Southeast Asian traditional medicine [11,13]. The leaves of *V. negundo* are particularly rich in bioactive secondary metabolites, including flavonoids, phenolic acids, iridoid glycosides, and terpenoids [11,13,14]. Previous studies using GC–MS and HPLC analyses have demonstrated antibacterial activity against both Gram-positive and Gram-negative bacteria through membrane disruption, biofilm inhibition, and virulence factor suppression [[14], [15], [16]]. Notably, flavonoids and phenolic acids characteristic of *V. negundo* have been reported to interfere with quorum sensing and suppress virulence-associated gene expression in pathogenic Enterobacteriaceae, suggesting their potential relevance against Stx-expressing STEC strains [9,16]. Despite this promising phytochemical profile, comprehensive metabolite characterization of *V. negundo* leaf extracts using high-resolution tandem mass spectrometry and systematic evaluation of their activity against STEC O157:H7 via Stx protein expression remain lacking.

To address this knowledge gap, phytochemical investigation using UPLC-MS/MS offers a powerful approach for comprehensive metabolite annotation in complex plant matrices. Feature-based molecular networking (FBMN) implemented on the GNPS (Global Natural Products Social Molecular Networking) platform organizes MS/MS spectra into chemically related molecular families based on cosine similarity scoring, enabling rapid dereplication of known metabolites and structural annotation of novel analogs [17,18]. Compared with conventional HPLC-DAD or GC–MS approaches previously applied to *V. negundo* [14,15], UPLC-MS/MS integrated with GNPS provides superior resolution, sensitivity, and annotation coverage without requiring exhaustive compound isolation, making it particularly well suited for untargeted profiling of complex leaf extracts [19,20].

Therefore, the present study aimed to: (i) identify and annotate secondary metabolites in *V. negundo* leaf extracts using UPLC-MS/MS integrated with GNPS-based molecular networking; (ii) evaluate the crude extract's potential in formulating biodegradable films for food safety applications; and (iii) identify targeted anti-Stx lead compounds through molecular docking, dynamics simulations, and ADMET profiling to support future therapeutic development.

## 2 Materials and methods

### 2.1 Plant material and extraction procedure

Leaves of *V. negundo* were sourced from Can Tho City, Vietnam, and botanical identification was performed by Dr. Pham Ha Thanh Tung (Phenikaa University, Vietnam). Before extraction, damaged leaves were discarded, and the remaining material was thoroughly washed, air-dried, and mechanically pulverized to yield a homogeneous dry powder. A total of 0.5 kg of powder was soaked in 96° ethanol for 3 days, and the crude extract was treated with anhydrous Na_2_SO_4_ and concentrated by rotary evaporation. The maceration process was performed three times in total to maximize compound recovery, yielding an extraction efficiency of approximately 12% and a moisture content of approximately 4.6–5.0%. After being weighed, the crude extract was stored at 4 °C.

### 2.2 HRESI-qTOF ms/ms sample measurement

The chemical profile of the ethanolic extract was characterized by high-resolution electrospray ionization quadrupole time-of-flight tandem mass spectrometry (HRESI-qTOF MS/MS). Chromatographic separation was performed using a Waters Acquity UPLC system coupled to a Waters Xevo G2 QTOF mass spectrometer (Waters Co., Manchester, UK), with data acquisition in negative- and positive-ion modes. Analytes were resolved on a Waters Acquity UPLC BEH C18 column (150 mm × 2.1 mm, 1.7 µm particle size) using a binary gradient of acetonitrile and water (ranging from 10:90 to 90:10, v/v) over a 24-minute run. The mobile phase was delivered at a flow rate of 300 µL/min, and the sample injection volume was set to 2.0 µL. The autosampler compartment and column oven were thermostatically controlled at 15 °C and 40 °C, respectively.

Electrospray ionization (ESI) source conditions were optimized as follows: capillary voltage, 2.5 kV; cone voltage, 40 V; source temperature, 120 °C; desolvation gas temperature, 350 °C; cone gas flow rate, 50 L/h; and desolvation gas flow rate, 800 L/h. Mass spectra were acquired at a rate of 0.2 s per spectrum. To maintain mass accuracy and measurement reproducibility throughout the analytical run, centroid data were continuously corrected using a reference lock-mass ion introduced via the LockSpray interface.

### 2.3 Molecular networking and spectral library search

Molecular networking analysis was performed using the Feature-Based Molecular Networking (FBMN) workflow implemented on the GNPS platform (https://gnps.ucsd.edu) [19]. Raw mass spectrometry data were processed in MZmine2, and the resulting feature tables and MS/MS spectra were uploaded to GNPS for network construction. Before network generation, spectral data were preprocessed to improve data quality. Fragment ions within ±17 Da of the precursor *m/z* were excluded to eliminate artifacts from neutral losses of water and ammonia. Additionally, each spectrum was simplified by retaining only the six most intense fragment ions within any ±50 Da window. Precursor ion mass tolerance and MS/MS fragment ion tolerance were both set to 0.02 Da. Spectral similarity between network nodes was assessed using the cosine score metric. Edges connecting two nodes were retained only when the cosine similarity exceeded 0.65, and at least four fragment ions were matched between the two spectra. To prevent overly large and uninterpretable clusters, the maximum allowable size of any molecular family was capped at 100 nodes. Mass accuracy was evaluated as the difference between the observed precursor *m/z* and the precursor *m/z* of the matched GNPS library entry, expressed in Da (MassDiff) and in ppm. Beyond the search tolerance, library matches were retained only when this mass error was within 10 ppm.

Compound annotation was performed by matching network spectra against the GNPS reference spectral libraries [18] using the same spectral filtering criteria described above. Library matches were accepted only when the cosine score was greater than 0.65 and at least 3 shared peaks were identified. The resulting molecular networks were visualized in Cytoscape [20]. The complete molecular networking job is publicly accessible at https://gnps.ucsd.edu/ProteoSAFe/status.jsp?task=62137e86beed4a15b32385f222244e5f for negative mode FBMN and at https://gnps.ucsd.edu/ProteoSAFe/status.jsp?task=6762ee2a784345edafcccf47816ae5bf for positive mode FBMN.

### 2.4 Molecular docking study

To evaluate ligand-Shiga toxin binding interactions *in silico*, molecular docking was performed using the AutoDock Vina algorithm within PyRx [21]. Two-dimensional molecular structures were initially constructed using ChemDraw version 16 and subsequently geometrically optimized using the semi-empirical method implemented in Gaussian 09. The three-dimensional coordinates for Stx1 and Stx2 proteins were retrieved from the Protein Data Bank (PDB ID: 1QNU and 3MXG, respectively) [22,23]. For the docking calculations, search grids were established with the following specifications: Stx1 protein centered at coordinates (X: 13.36, Y: 15.15, Z: 10.79) with grid dimensions of (X: 21.10, Y: 21.40, Z: 20.22) Å, while Stx2 protein was centered at (X: 8.67, Y: 49.09, Z: 0.08) with corresponding dimensions of (X: 18.82, Y: 22.33, Z: 24.13) Å. Docking calculations were performed with an exhaustiveness value of 8, generating up to 9 binding poses per ligand within an energy range of 3 kcal/mol relative to the top-scoring pose. The molecular interaction patterns obtained from docking simulations were analyzed and presented using DS Visualizer software. Hydrogen bond geometric parameters were determined using UCSF Chimera software. All hydrogen-bond distances are reported as hydrogen-acceptor separations.

### 2.5 Molecular dynamics simulation

The protein-ligand binding complexes obtained from docking studies were subsequently subjected to molecular dynamics (MD) simulations employing the NAnoscale Molecular Dynamics (NAMD2) computational platform [24]. Structural parameterization and force-field assignment for both protein and ligand components were performed using the CHARMM-GUI web interface with the CHARMM36m force field. The molecular systems were immersed in explicit TIP3P water models, and ionic strength was maintained at physiological conditions by incorporating 0.15 M sodium chloride for charge neutralization. After initial energy minimization, each system underwent a 100 ps equilibration phase to achieve structural stability. Production simulations were then performed under isothermal-isobaric (NPT) ensemble conditions at 310 K for 100 ns. Trajectory analysis was performed using the Visual Molecular Dynamics (VMD 1.9.3) software package to evaluate protein backbone conformational stability via root-mean-square deviation (RMSD) calculations and to quantify intermolecular hydrogen-bonding interactions [25]. Hydrogen bonds along the trajectory were identified using the default VMD geometric criteria (donor-acceptor distance ≤ 3.5 Å, donor-hydrogen-acceptor angle cutoff 30°). Note that the trajectory analysis used the donor-acceptor convention, whereas the docking distances in Section 2.4 were hydrogen-acceptor separations; the two differ by the length of the D–H covalent bond and were therefore reported separately.

### 2.6 Preparation of biodegradable films

Starch-based films were prepared by dispersing 5.0 g of sweet potato starch in 50 mL of distilled water, then adding 10% w/w glycerol as a plasticizer. The suspension was gelatinized under continuous magnetic stirring at 75 °C for 25 min until a uniform gel formed. *V. negundo* leaf extract, which had been defatted and dechlorophyllated with *n*-hexane, and citric acid were then incorporated at 10% (w/w) and 1.5% (w/w), respectively. The film-forming solution was cast into molds and dried at ambient temperature for 48 h, followed by a short drying step at 50 °C for 10 min to facilitate film release. These films were stored in sealed containers with desiccant, and the film containing glycerol, *V. negundo* leaf extract and citric acid was measured for tensile strength according to the modified ASTM D882 standard [26].

### 2.7 Antibacterial activity assay using the resazurin-based method

*E. coli* O157:H7 strain ATCC 43,888 was cultured in Tryptone Soya Broth (TSB) and incubated under aerobic conditions at 37 °C overnight. For the experiments, the bacteria were inoculated into fresh media and adjusted to 5 × 10^5^ CFU/mL. The antibacterial assay was performed in 96-well plates. Briefly, 50 µL of serially diluted plant extracts or a positive control (Tetracycline, Himedia) was added to each well, followed by 50 µL of the bacterial suspension, bringing the total volume to 100 µL per well. The plates were wrapped and incubated at 32 °C for 24 h. Following this primary incubation, 10 µL of resazurin indicator solution (0.015%w/v) was added to each well. The plates were then incubated for an additional 2–4 h in the dark. The minimum inhibitory concentration (MIC) was determined by observing the color transition in the wells. All experiments were performed in triplicate (n = 3). The lowest concentration of the extract that prevented the blue resazurin dye from being reduced to pink resorufin by bacterial metabolic activity was recorded as the MIC [27].

### 2.8 Antibacterial activity assay using standard plate count

Antibacterial activity of the biodegradable films was assessed using a modified Standard Plate Count (droplet method). *E. coli* was cultured in TSB for 24 h, then subcultured with shaking for 1 hour before exposure to five treatments: bacteria alone, bacteria with commercial PP plastic film, bacteria with glycerol-containing film, bacteria with film containing glycerol and extract, and bacteria with film containing glycerol, extract, and citric acid. After 1 hour of shaking, each suspension was serially diluted (10^–1^ to 10^–5^) in sterile 0.9% saline. Aliquots of 10 µL from each dilution were spotted in triplicate onto TSA plates and incubated at 32 °C for 12 h. Surviving colonies were enumerated and bacterial density (CFU/mL) calculated as: CFU/mL = colony count × dilution factor × 1000/V, where V = 10 µL. Each treatment was performed in three independent experiments. Statistical comparisons versus the bacterial control were performed using one-way ANOVA with Dunnett’s test [28].

### 2.9 Biodegradability test

Commercial PP plastic and biodegradable films measuring 2.5 × 2.5 cm were buried in soil at 4 − 6 cm depth at room temperature. Soil moisture was maintained by daily surface watering. The films were observed for degradation to evaluate their disintegration during 28 days of the experiment [29].

### 2.10 ADMET profiling

Absorption, distribution, metabolism, excretion, and toxicity (ADMET) properties of compound 8 were predicted using the SwissADME web server [30] and pkCSM [31], both accessed with default parameters. Physicochemical descriptors (LogP, TPSA, hydrogen bond donors/acceptors), pharmacokinetic properties (gastrointestinal absorption, blood–brain barrier permeability), and predicted toxicity endpoints were retrieved directly from these platforms.

## 3 Results and discussion

### 3.1 Molecular networking of V. negundo

UPLC-qTOF-MS/MS analysis of the ethanolic leaf extract of *V. negundo* produced well-resolved base peak chromatograms in both negative and positive ESI modes (Fig. 1). In negative mode, ion signals accumulated between 2 and 15 min, a retention window characteristic of polar phenolic glycosides, organic acids, and iridoid glucosides. The positive mode chromatogram extended to approximately 21 min, during which less polar constituents − terpenoids, fatty acid derivatives, and alkaloids − were detected with acceptable sensitivity. This dual-polarity acquisition was deliberately chosen because many plant secondary metabolites exhibit a strong ionization preference for one polarity over the other, and restricting analysis to a single polarity would have introduced systematic gaps in coverage [17,18].

**Fig. 1:**
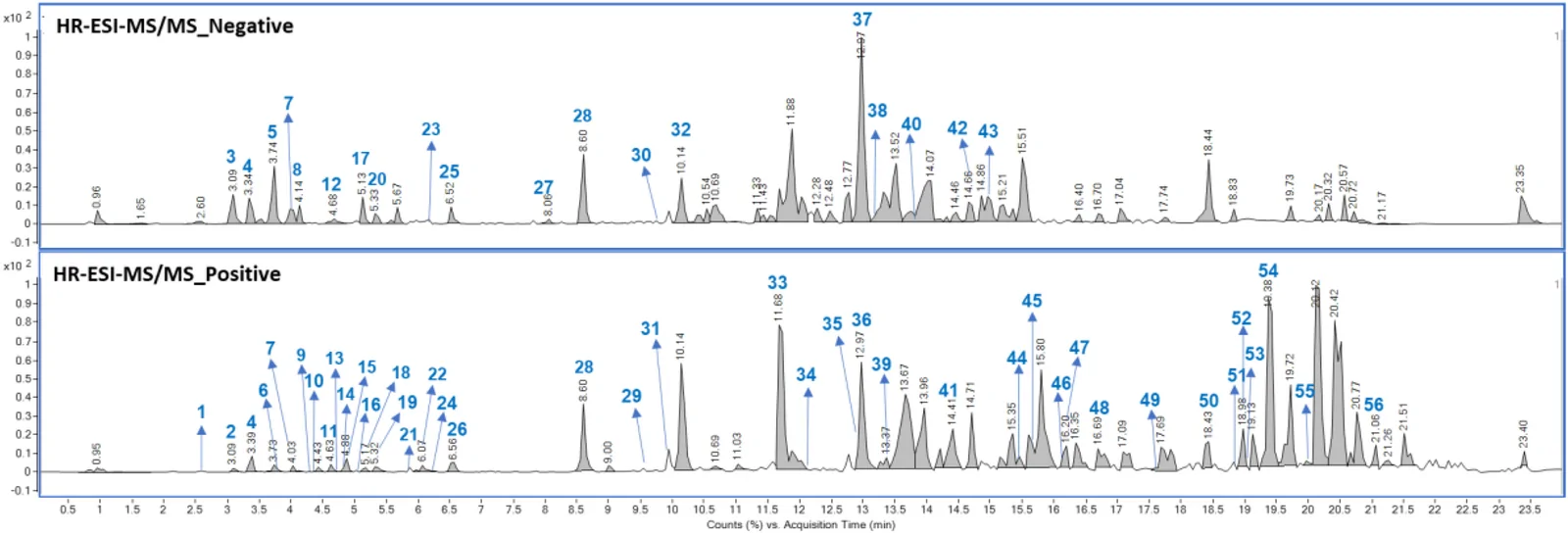
Base peak chromatogram obtained by UPLC-qTOF-MS/MS of *V. negundo* leaf extract in negative and positive modes, with the putatively annotated compounds obtained from GNPS.

For the annotation step, we applied FBMN on the GNPS platform [19], which, to our knowledge, has not previously been reported for *V. negundo* leaf extracts. Raw data were processed in MZmine2 to generate a feature table that preserves retention time and peak intensity information for each detected ion. Before network construction, spectral data underwent two preprocessing steps: fragment ions within ±17 Da of the precursor *m/z* were removed to eliminate water- and ammonia-loss artifacts, and each spectrum was simplified by retaining only the six most intense fragment ions per ±50 Da window. Network edges were drawn between pairs of nodes whose cosine similarity exceeded 0.65, and that shared at least four matched fragment ions, with precursor and MS/MS fragment tolerances both set to 0.02 Da. To maintain network interpretability, each molecular family was capped at 100 nodes.

The resulting networks (Figs. 2, 3, S1 and S2) organized the detected features into structurally coherent clusters. In negative mode (Fig. 2), four principal groupings were observed: flavonoid aglycones (nodes 23, 28, 30, 32), flavonoid *C*- and *O*-glycosides (nodes 4, 7), iridoid glycosides (nodes 3, 12, 17), and naphthalene-type sesquiterpenoids (node 37). In positive mode (Fig. 3), additional clusters resolved for fatty acid derivatives and glycolipids (nodes 35, 50, 54), isoquinoline alkaloids (nodes 18, 22, 26), diterpenoids (nodes 47, 49, 53, 55), and a second naphthalene-type sesquiterpenoid group (node 39), with several nodes shared across both polarity networks, reinforcing those assignments. Orange nodes in the figures correspond to compounds matched directly to the GNPS spectral library, while blue nodes represent putative structural analogs inferred by fragmentation similarity within the same cluster. This propagation of annotation from confirmed library hits to neighboring, unmatched nodes is a central advantage of FBMN: it provides chemical context for minor or unknown congeners without requiring their isolation [19,20].

**Fig. 2:**
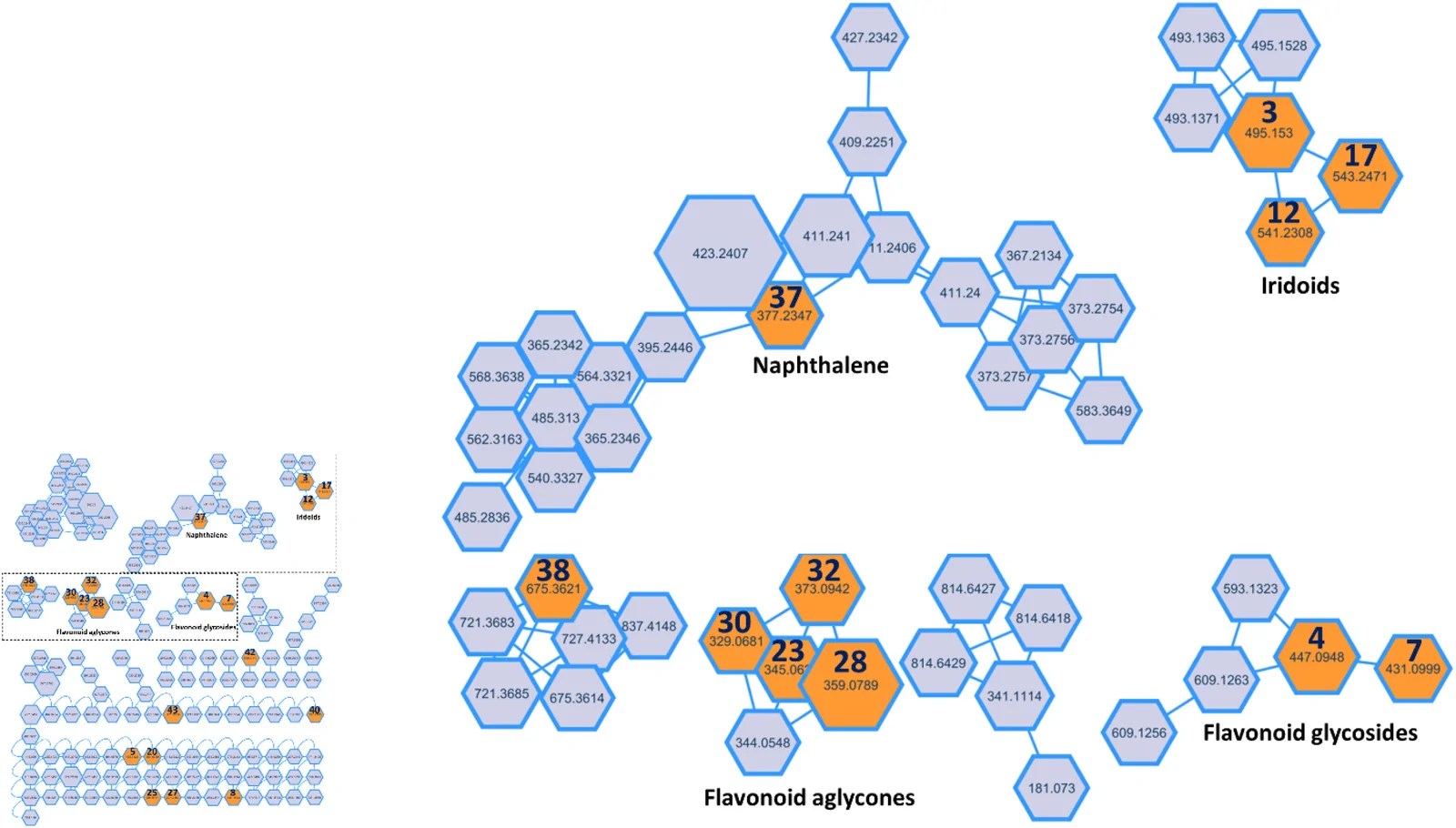
Feature-based Molecular networking of *V. negundo* leaf extract in negative mode.

**Fig. 3:**
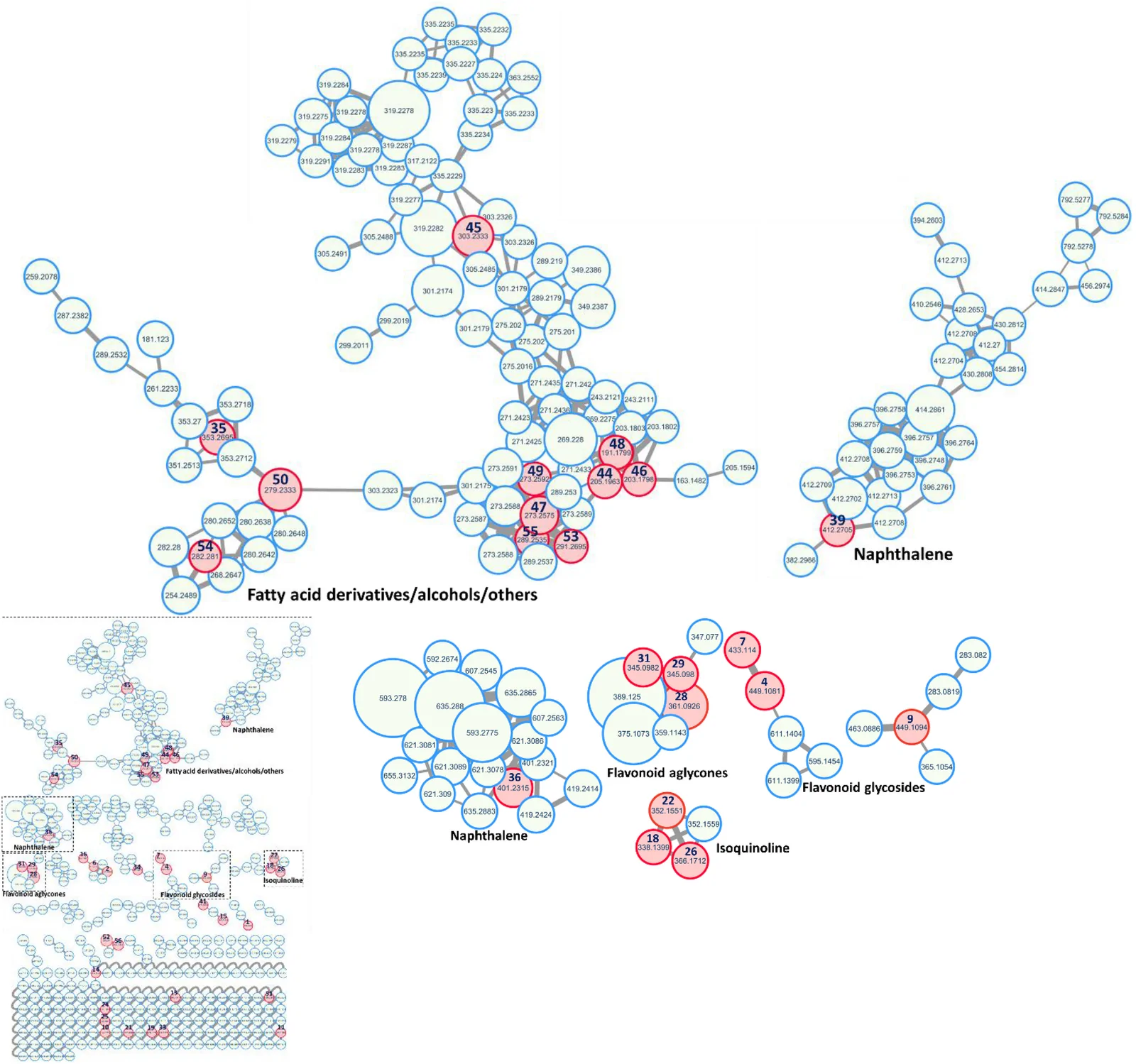
Feature-based Molecular networking of *V. negundo* leaf extract in positive mode.

Applying library matching criteria (cosine score >0.65, minimum three shared peaks, mass error < 10 ppm), 56 metabolites were putatively annotated (Table 1, Fig. 4). Across the retained annotations, mass errors ranged from 0.20 to 8.35 ppm, and all annotations fell within the 10 ppm acceptance window. The annotated compounds comprised: phenolic acids (compounds 1, 2, 6), flavonoid aglycones (compounds 23, 25, 28–33), flavonoid *C*/*O*-glycosides (compounds 4, 7, 9, 20), iridoid glycosides (compounds 3, 5, 8, 12, 17), oxylipins and fatty acid derivatives (compounds 27, 40, 41, 50, 51), glycolipids (compound 38, 42), isoquinoline and miscellaneous alkaloids (compounds 10, 14, 18, 19, 21, 22, 24, 26), triterpenoids and diterpenoids (compounds 34, 43, 47, 49, 53), and naphthalene-type sesquiterpenoids (compounds 36, 37, 39).

**Table 1:** Putative compounds of *V. negundo* leaf extract based on the HRESI-qTOF-MS/MS in negative and positive modes.

Comp. No.	*m/z*	Retention time (min)	Adduct	Compound name	MassDiff (Da)	MZError (ppm)
**1**	**139.0394**	2.59	[M+H]^+^	3-Hydroxybenzoic acid	0.0004	2.85
**2**	**121.0289**	3.09	[M-H_2_O+H]^+^	Salicylic acid	0.0009	7.44
**3**	**495.1530**	3.09	[M-H]^-^	(1*S*,4a*S*,7*S*,7a*S*)-7‑hydroxy-7-methyl-1-[(2*S*,3*R*,4*S*,5*S*,6*R*)-3,4,5-trihydroxy-6-[(4-hydroxybenzoyl)oxymethyl]oxan-2-yl]oxy-4a,5,6,7a-tetrahydro-1H-cyclopenta[c]pyran-4-carboxylic acid	0.0020	4.07
**4**	**447.0948**	3.34	[M-H]^-^	Homoorientin (Isoorientin)	0.0018	4.03
**4**	**449.1081**	3.39	[M+H]^+^	Homoorientin (Isoorientin)	0.0001	0.20
**5**	**465.1425**	3.74	[M-H]^-^	Agnuside	0.0025	5.31
**6**	**147.0444**	3.83	[M-H_2_O+H]^+^	*p*-Coumaric acid	0.0004	2.70
**7**	**433.1140**	4.03	[M+H]^+^	Isovitexin	0.0030	6.98
**7**	**431.0999**	4.04	[M-H]^-^	Isovitexin	0.0019	4.46
**8**	**537.1633**	4.14	[M-H]^-^	(1*S*,4a*S*,7*S*,7a*S*)-1-[(2*S*,3*R*,4*S*,5*S*,6*R*)-6-[[(*E*)-3-(3,4-Dihydroxyphenyl)prop‑2-enoyl]oxymethyl]−3,4,5-trihydroxyoxan-2-yl]oxy-7‑hydroxy-7-methyl-4a,5,6,7a-tetrahydro-1H-cyclopenta[c]pyran-4-carboxylic acid	0.0023	4.32
**9**	**449.1094**	4.28	[M+H]^+^	Luteolin 4′-*O*-glucoside	0.0014	3.13
**10**	**342.1710**	4.38	[M+H]^+^	Corypalmine	0.0010	2.85
**11**	**132.0810**	4.63	[M+H]^+^	Skatole	0.0003	2.27
**12**	**541.2308**	4.68	[M-H]^-^	7-Hydroxy-1-({2-*O*-[(2*E*,6*E*)-8‑hydroxy-2,6-dimethyl-2,6-octadienoyl]-beta-d-glucopyranosyl}oxy)-7-methyl-1,4a,5,6,7,7a-hexahydrocyclopenta[c]pyran-4-carboxylic acid	0.0018	3.27
**13**	**499.1241**	4.73	[M-H_2_O+H]^+^	(3*R*,5*R*)-3,5-bis[[(*E*)-3-(3,4-dihydroxyphenyl)prop‑2-enoyl]oxy]−1,4-dihydroxycyclohexane-1-carboxylic acid	0.0011	2.26
**14**	**354.1354**	4.83	[M+H]^+^	Protopine	0.0014	3.96
**15**	**197.1185**	4.88	[M+H]^+^	2(4H)-Benzofuranone, 5,6,7,7a-tetrahydro-6‑hydroxy-4,4,7a-trimethyl-, (6*S*,7a*R*)-	0.0015	7.59
**16**	**146.0607**	5.03	[M+H]^+^	Indole-3-carboxyaldehyde	0.0003	2.09
**17**	**543.2471**	5.13	[M-H]^-^	(1*S*,4a*S*,7*S*,7a*S*)-7-(((E)-8‑hydroxy-2,6-dimethyloct-2-enoyl)oxy)-7-methyl-1-(((2*S*,3*R*,4*S*,5*S*,6*R*)-3,4,5-trihydroxy-6-(hydroxymethyl)tetrahydro-2H-pyran-2-yl)oxy)-1,4a,5,6,7,7a-hexahydrocyclopenta[c]pyran-4-carboxylic acid	0.0021	3.82
**18**	**338.1399**	5.17	M^+^	Jatrorrhizine	0.0009	2.62
**19**	**356.1874**	5.32	[M+H]^+^	1,2,9,10-tetramethoxy-6-methyl-5,6,6a,7-tetrahydro-4H-dibenzo[*de,g*]quinoline	0.0014	3.94
**20**	**461.0739**	5.33	[M-H]^-^	(2*S*,3*S*,4*S*,5*R*,6*S*)-6-[2-(3,4-Dihydroxyphenyl)-5‑hydroxy-4-oxochromen-7-yl]oxy-3,4,5-trihydroxyoxane-2-carboxylic acid	0.0009	1.99
**21**	**370.2026**	5.87	[M+H]^+^	Corydaline	0.0026	7.01
**22**	**352.1551**	6.07	M^+^	Palmatine	0.0001	0.26
**23**	**345.0622**	6.12	[M-H]^-^	2-(3,4-Dihydroxyphenyl)-5,7-dihydroxy-6,8-dimethoxychromen-4-one	0.0002	0.53
**24**	**314.1394**	6.17	[M+H]^+^	(*E*)-3-(4‑hydroxy-3-methoxyphenyl)-*N*-[2-(4-hydroxyphenyl)ethyl]prop‑2-enamide	0.0008	2.53
**25**	**287.0567**	6.51	[M+H]^+^	Luteolin	0.0007	2.45
**25**	**285.0424**	6.52	[M-H]^-^	Luteolin	0.0024	8.35
**26**	**366.1712**	6.56	M^+^	Dehydrocorydaline	0.0022	6.00
**27**	**327.2189**	8.06	[M-H]^-^	(10*E*,15*E*)-9,12,13-trihydroxyoctadeca-10,15-dienoic acid	0.0009	2.80
**28**	**361.0926**	8.60	[M+H]^+^	Jaceidin	0.0006	1.61
**28**	**359.0789**	8.60	[M-H]^-^	Jaceidin	0.0019	5.27
**29**	**345.0980**	9.54	[M+H]^+^	Eupatorin	0.0020	5.84
**30**	**329.0681**	9.75	[M-H]^-^	(2*Z*)-4,6-dihydroxy-2-[(4‑hydroxy-3,5-dimethoxyphenyl)methylidene]−1-benzofuran-3-one	0.0011	3.43
**31**	**345.0982**	9.94	[M+H]^+^	Santin	0.0012	3.54
**32**	**373.0942**	10.14	[M-H]^-^	Casticin	0.0012	3.27
**33**	**799.2221**	11.68	[2M+Na]^+^	Artemisetin	0.0011	1.37
**34**	**471.3472**	12.13	[M-H_2_O+H]^+^	Asiatic acid	0.0002	0.45
**35**	**353.2695**	12.92	[M+H]^+^	Monolinolenin (9c,12c,15c)	0.0005	1.38
**36**	**401.2315**	12.97	[M+Na]^+^	5-[(4*S*,8a*S*)-4-acetyloxy-2,5,5,8a-tetramethyl-3-oxo-4a,6,7,8-tetrahydro-4H-naphthalen-1-yl]−3-methylpentanoic acid	0.0015	3.73
**37**	**377.2347**	12.97	[M-H]^-^	[(1*R*,3*R*,4*R*,4a*S*)-4‑hydroxy-3,4a,8,8-tetramethyl-4-[2-(5-oxo-2H-furan-3-yl)ethyl]−2,3,5,6,7,8a-hexahydro-1H-naphthalen-1-yl] acetate	0.0017	4.53
**38**	**675.3621**	13.27	[M-H]^-^	DGMG 18:3	0.0031	4.61
**39**	**412.2705**	13.37	[M+NH_4_]^+^	2-Hydroxy-2′',5′',5′',8a''-tetramethyl-5-oxododecahydro-2′'H-dispiro[furan-3,2′-furan-5′,1′'-naphthalen]−4′'-yl acetate	0.0015	3.63
**40**	**293.2135**	13.82	[M-H]^-^	13-HOTrE	0.0015	5.10
**41**	**275.2019**	14.41	[M-H_2_O+H]^+^	9-OxoOTrE	0.0019	6.88
**42**	**559.3149**	14.66	[M+FA-H]^-^	9,12,15-Octadecatrienoic acid, 3-(hexopyranosyloxy)-2-hydroxypropyl ester, (9*Z*,12*Z*,15*Z*)-	0.0029	5.13
**43**	**471.3508**	14.96	[M-H]^-^	Corosolic acid	0.0028	5.94
**44**	**205.1963**	15.45	[M-H_2_O+H]^+^	Nerolidol	0.0013	6.32
**45**	**303.2333**	15.75	[M-H_2_O+H]^+^	KU036–9–2a	0.0013	4.33
**46**	**203.1798**	16.15	[M-H_2_O+H]^+^	1-Methyl-4-methylidene-7-(propan-2-yl)-1,2,3,3a,4,5,6,8a-octahydroazulen-1-ol	0.0008	3.91
**47**	**273.2575**	16.20	[M-H_2_O+H]^+^	Manool	0.0005	1.79
**48**	**191.1799**	16.69	[M-2H_2_O+H]^+^	2,4,7,9-Tetramethyl-5-decyne-4,7-diol	0.0001	0.48
**49**	**273.2592**	17.64	[M+H]^+^	Cembrene	0.0012	4.36
**50**	**279.2333**	18.43	[M+H]^+^	Linolenic acid	0.0013	4.70
**51**	**305.2480**	18.78	[M-H_2_O+H]^+^	15-OxoEDE	0.0010	3.20
**52**	**256.2640**	18.98	[M+H]^+^	Palmitamide	0.0010	3.93
**53**	**291.2695**	19.03	[M+H]^+^	2-Penten-1-ol, 5-[(1*S*,4a*S*,8a*S*)-decahydro-5,5,8a-trimethyl-2-methylene-1-naphthalenyl]−3-methyl-, (2*E*)-	0.0015	5.13
**54**	**282.2810**	19.38	[M+H]^+^	9-Octadecenamide, (*Z*)	0.0010	3.57
**55**	**289.2535**	19.97	[M-H_2_O+H]^+^	KU036–10	0.0005	1.69
**56**	**284.2956**	21.06	[M+H]^+^	Octadecanamide	0.0004	1.40

MassDiff, difference between the observed precursor *m/z* and that of the matched GNPS library entry (Da); MZError, the same difference expressed in ppm.

**Fig. 4:**
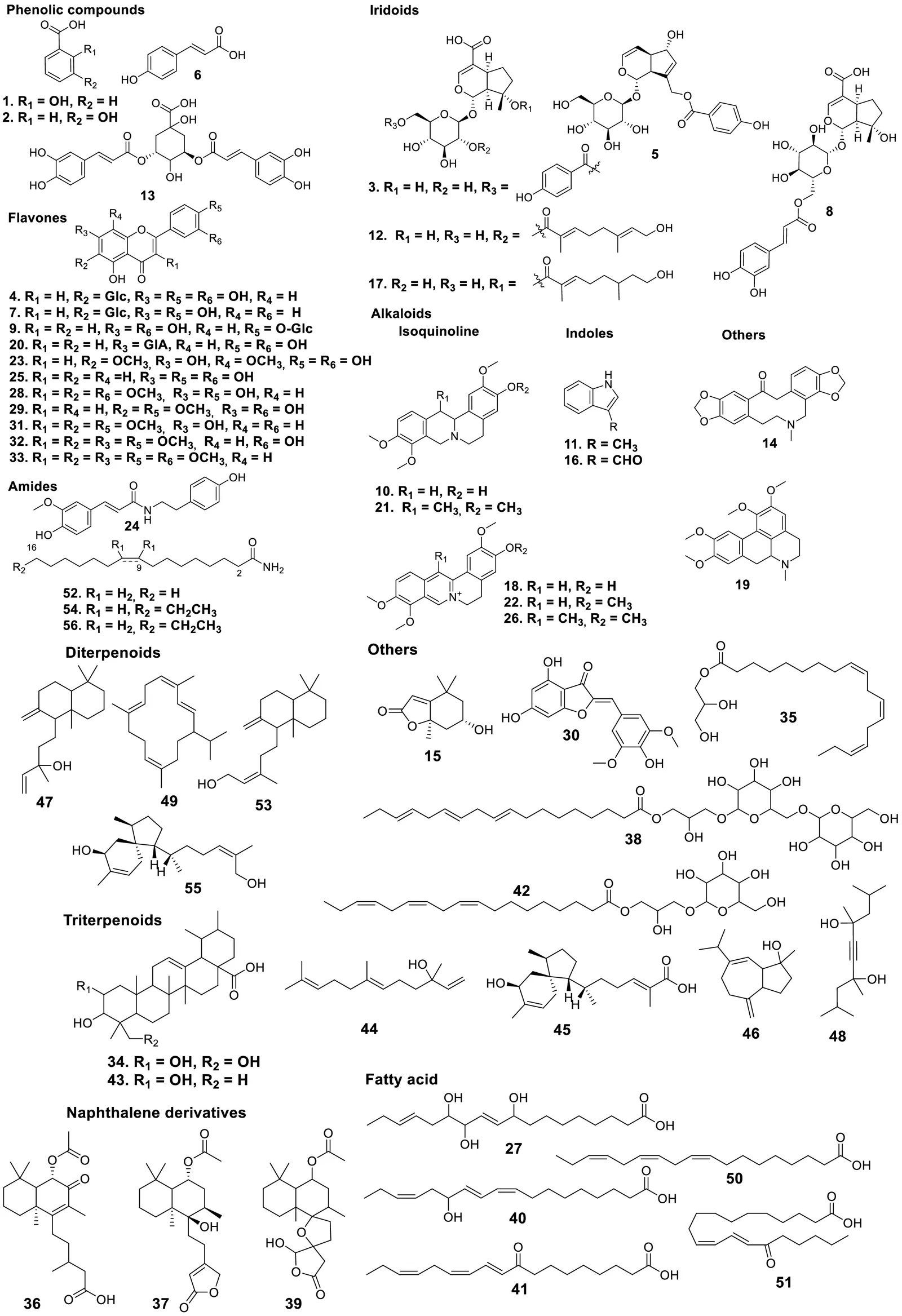
Putative structures from *V. negundo* leaf extract obtained from GNPS.

Among phenolic metabolites, the *C*-glycosyl flavones homoorientin (compound 4) and isovitexin (compound 7) were detected in both ionization modes and assigned with high confidence, consistent with their documented occurrence in *Vitex* species [13,16]. The flavonoid aglycone cluster was anchored by luteolin (compound 25) and its polymethoxylated derivatives casticin (compound 32), jaceidin (compound 28), eupatorin (compound 29), santin (compound 31), and artemisetin (compound 33). The accumulation of polymethoxyflavones is a phytochemical hallmark of the genus *Vitex*, and these compounds have been linked to stronger membrane-disruptive effects than their less-substituted hydroxylated counterparts, owing to increased lipophilicity and greater membrane partitioning [32]. The iridoid glycoside cluster was anchored by agnuside (compound 5), widely regarded as a chemotaxonomic marker of *V. negundo* [33], together with three structurally related iridoid esters (compounds 3, 8, 12), whose configuration differences are captured in the network as closely spaced nodes with high pairwise cosine scores. Compound 8 was putatively annotated based on GNPS spectral library matching (cosine = 0.96, 21 shared fragment peaks, mass error = 4.32 ppm), corresponding to a Metabolomics Standards Initiative (MSI) level 2 annotation.

Positive-mode networking resolved a cluster of protoberberine isoquinoline alkaloids − jatrorrhizine (compound 18), palmatine (compound 22), dehydrocorydaline (compound 26), corypalmine (compound 10), and corydaline (compound 21) − alongside protopine (compound 14), a benzylisoquinoline. These alkaloids are not commonly reported in *V. negundo*, and their detection here is worth treating with appropriate caution: spectral library matches for protoberberines can occasionally arise from cross-reactivity with structurally related flavonoids at low cosine score thresholds. Confirmation by authentic standards would be desirable before drawing pharmacological conclusions. The previous study suggested trace alkaloid signals in *V. negundo* leaf extracts analyzed by 2D-LC-MS/MS, lending some plausibility to these annotations [16].

The fatty acid region of the positive-mode network (Fig. 3) contained linolenic acid (compound 50) together with its oxygenated metabolites 13-HOTrE (compound 40) and a trihydroxyoctadecadienoic acid derivative (compound 27). The co-occurrence of free polyunsaturated fatty acids and their hydroperoxy/hydroxy derivatives points to active lipoxygenase-type oxygenation in the leaf tissue [34]. Notably, compound 38 (DGMG 18:3, digalactosylmonoacylglycerol) was annotated as a glycolipid rather than a naphthalene derivative; its position in the positive-mode fatty acid cluster (Fig. 3) is therefore structurally consistent with its ester-linked α-linolenic acid moiety [35]. Triterpenoids asiatic acid (compound 34) and corosolic acid (compound 43), both ursane-type pentacyclics, were annotated alongside three diterpenoids (compounds 47, 49, 53) and three naphthalene-type sesquiterpenoids (compounds 36, 37, 39), the last group representing structurally unusual metabolites for the Lamiaceae, whose biosynthetic origin in *Vitex* has not been clarified.

Compared with previous targeted analyses of *V. negundo* leaves, which typically putatively annotated 10–25 compounds when relying on GC–MS profiling of volatile fractions or HPLC-DAD monitoring of major polyphenols [14,15], the untargeted FBMN approach employed here annotated more than twice as many metabolites and, more usefully, placed them in a structural context that facilitates chemical interpretation. Earlier fingerprinting studies provided little information on the minor constituents − iridoid esters, oxylipins, glycolipids, and naphthalene sesquiterpenoids − that are now visible in the networks. From a food safety perspective, the simultaneous detection of luteolin, casticin, and iridoid glycosides is of particular interest, since flavonoids of this type have been shown to attenuate quorum sensing and downregulate virulence-associated gene expression in enterohemorrhagic *E. coli*, an effect documented for structurally similar compounds at sub-inhibitory concentrations [9]. The oxygenated linolenic acid derivatives add a further dimension worth investigating, as hydroxy fatty acids have been reported to perturb bacterial membrane fluidity and modulate stress responses in Gram-negative pathogens [36].

### 3.2 In vitro antibacterial activity of V. negundo extract against *E. coli* O157:H7

The crude *V. negundo* extract inhibited *E. coli* O157:H7 growth at an MIC of 4 mg/mL, a value consistent with the range previously reported for this species against enteric Gram-negative pathogens (1.25–12.8 mg/mL, depending on solvent system and test strain) [37,38]. The positive control, tetracycline, yielded an MIC of 8 µg/mL under the same conditions. The 500-fold difference between these values is expected rather than indicative of weak activity: tetracycline is a single purified molecule optimized for ribosomal binding, whereas the *V. negundo* extract is a complex mixture in which bioactive constituents represent only a minor fraction of the total dried mass. The observed MIC, therefore, reflects the cumulative contribution of multiple active compounds diluted within a crude extract containing many inactive components.

The chemical map produced by FBMN in Section 3.1 narrows the likely candidates. Three metabolite classes most plausibly account for the inhibitory activity: the polymethoxylated flavonoids (luteolin, casticin, jaceidin, eupatorin), the iridoid glycosides centered on agnuside, and the oxygenated linolenic acid derivatives. Each has been independently associated with membrane disruption, interference with quorum sensing, or downregulation of virulence-related genes in enterohemorrhagic *E. coli* [9,16,36]. Their simultaneous occurrence in the same extract provides a coherent rationale for the moderate but reproducible activity recorded.

Another consideration concerns the mechanism of clinical injury in STEC O157:H7. The pathogenicity of this bacterium does not arise from intestinal colonization *per se*, but from the Shiga toxin it produces [2,3]. This distinction matters therapeutically, because bactericidal antibiotics are generally inadvisable in this setting: cell lysis activates the SOS response and can amplify circulating Stx even at a low MIC in vitro [4]. Tetracycline, as the positive control in this study and generally regarded as bacteriostatic, avoids this acute lysis risk and inhibited STEC O157:H7 growth far more potently than the *V. negundo* extract (MIC 8 µg/mL vs. 4 mg/mL). However, like most conventional antibiotics, tetracycline acts primarily by suppressing bacterial growth and does not directly neutralize Shiga toxin already released into circulation. Consequently, an agent capable of engaging the toxin itself − even one with only moderate antibacterial potency, such as the *V. negundo* extract − may offer greater therapeutic relevance for limiting toxin-mediated injury than a potent antibiotic that leaves circulating Stx unaddressed. The following section examines the individual *V. negundo* constituents that bind Stx at the molecular level.

### 3.3 Molecular docking between Shiga toxin proteins and the annotated metabolites

Molecular docking simulations were carried out using AutoDock Vina within the PyRx environment to assess the binding potential of the 56 putatively annotated *V. negundo* metabolites against the B-subunits of Shiga toxins Stx1 (PDB ID: 1QNU) and Stx2 (PDB ID: 3MXG). The B-subunit pentamer constitutes the cell-binding domain of both toxins. It is recognized as a validated pharmacological target for neutralizing Stx-mediated cytotoxicity, given that blocking the receptor-binding site prevents retrograde trafficking of the holotoxin to the endoplasmic reticulum, where the catalytic A-subunit exerts its ribosome-depurinating activity [22,23]. The Bridged-starfish inhibitor (STARFISH), a pentameric carbohydrate ligand in complex with the 1QNU protein [23], interacts with essential binding-pocket residues of the galactose-binding site, including Asn132, Arg133, Thr154, Gly162, Phe163, and Ser164. These results indicated that this is a potential target for inactivating Shiga toxin. In a previous report, an oxadiazole derivative (Oxa; corresponding to (2*E*)-3-(3,4-difluorophenyl)-2-(5-(hydroxy(pyridin-2-yl)methyl)-1,3,4-oxadiazol-2-yl)-*N*-methylacrylimidic acid [22]) was included as a positive control based on its documented in vitro antibacterial activity against *E. coli* O157:H7 and its previously reported *in silico* inhibitory profile against Stx1. Oxa achieved a calculated binding free energy of −7.05 kcal/mol against the Stx1 target, forming key hydrogen bonds with the catalytic residues Lys153, Thr154, and Ser164 of the B-subunit [22]. In the present work, re-docking of Oxa under our computational conditions yielded a binding energy of −6.9 kcal/mol (K*i* = 8.7 µM, inhibition constants throughout were derived from the docking score using the empirical relationship K*i* = exp(ΔG/RT) at 298 K [39]), with hydrogen bonds engaging the same residue triad (Lys153 (2.59 Å), Thr154 (2.19 Å), and Ser164 (1.79 Å)) and supplementary van der Waals contacts at Thr101, Thr119, Arg133, Asn155, and Gly162, along with π-type interactions (π-anion, amide-π stacked, π-alkyl) involving Phe130, Asn132, Ala156, Phe163, and Glu165 (Table S1; Table 2). The marginal difference of 0.15 kcal/mol relative to the published value falls well within the intrinsic scoring variability of AutoDock Vina. It confirms that our docking protocol reproduces the established binding mode of the reference compound [22]. This cross-validation provides a reliable benchmark for objectively assessing the phytochemical candidates.

**Table 2:** Molecular docking interaction types of Stx1 (PDB ID: 1QNU) with compound 8 and (2E)-3-(3,4-difluorophenyl)-2-(5-(hydroxy(pyridin-2-yl)methyl)-1,3,4-oxadiazol-2-yl)-N-methylacrylimidic acid.

Ligand	H-bond	van der Waals	Carbon Hydrogen Bond	π-π stacked/ π-π T-shaped/ amide-π stacked/ π-anion/ π -alkyl
**8**	THR119, THR121, ARG133, SER164	ASN115, GLU128, THR131, ASN132, ALA156, GLY160, PHE163	HIS158, GLY161, GLY162	PHE130
**Oxa**	LYS153, THR154, SER164	THR101, THR119, ARG133, ASN155, GLY162	-	PHE130, ASN132, ALA156, PHE163, GLU165

For the Stx1 target, the binding energies (ΔG) of the 56 putatively annotated compounds ranged from −3.7 to −6.9 kcal/mol, with K*i* values spanning from 8.7 µM to approximately 1933 µM (Table S1). These values reflect a pronounced spread in affinity, indicating that the structural diversity of the *V. negundo* metabolome translates into widely divergent recognition modes at the Stx1 active site. Among all tested compounds, the iridoid glycoside compound 8 (putatively annotated based on GNPS spectral library matching (cosine = 0.96, 21 shared fragment peaks, mass error = 4.32 ppm), corresponding to a Metabolomics Standards Initiative (MSI) level 2 annotation)) achieved the highest binding affinity, matching the Oxa control at ΔG = −6.9 kcal/mol (K*i* = 8.7 µM). Two flavonoid-derived compounds also performed strongly: isovitexin (compound 7, ΔG = −6.7 kcal/mol, K*i* = 12.2 µM) and luteolin-4′-*O*-glucoside (compound 9, ΔG = −6.7 kcal/mol, K*i* = 12.2 µM), followed by compounds 13 (ΔG = −6.6 kcal/mol), 20 (ΔG = −6.6 kcal/mol), 43 (corosolic acid, ΔG = −6.6 kcal/mol), and 3 (ΔG = −6.4 kcal/mol). Several additional compounds, including homoorientin (compound 4) and the triterpenoid asiatic acid (compound 34), also obtained ΔG values around −6.4 kcal/mol, suggesting that *V. negundo* has structurally diverse scaffolds with measurable Stx1 affinity.

Detailed analysis of the binding mode of compound 8 at the Stx1 active site (Table 2 and Fig. 5A, B) reveals a distinct interaction pattern relative to the Oxa control, despite the identical docking score. Compound 8 forms four conventional hydrogen bonds: two with the hydroxyl‑bearing Thr119 (3.36 Å) and Thr121 (2.82 Å), one with the guanidinium group of Arg133 (2.53 Å), and one with Ser164 (2.47 Å). The remaining network is composed of van der Waals contacts from Asn115, Glu128, Thr131, Asn132, Ala156, Gly160, and Phe163, carbon-hydrogen bonds from His158, Gly161, and Gly162, and a π-π stacking interaction with Phe130. By contrast, the Oxa control linked with Lys153, Thr154, and Ser164 through hydrogen bonding, with π-type interactions prevailing at the aromatic/aliphatic periphery (Table S1). The partial overlap in anchor residues (Ser164 and Arg133) between compound 8 and Oxa suggests that both ligands target the same galactose-binding pocket of the Stx1 B-subunit. These results indicate that compound 8 binds the polar hydroxyl‑rich segment of the cavity more extensively, consistent with its higher molecular polarity (log P = −0.4196, TPSA = 212.67 Å^2^) relative to the more lipophilic oxadiazole.

**Fig. 5:**
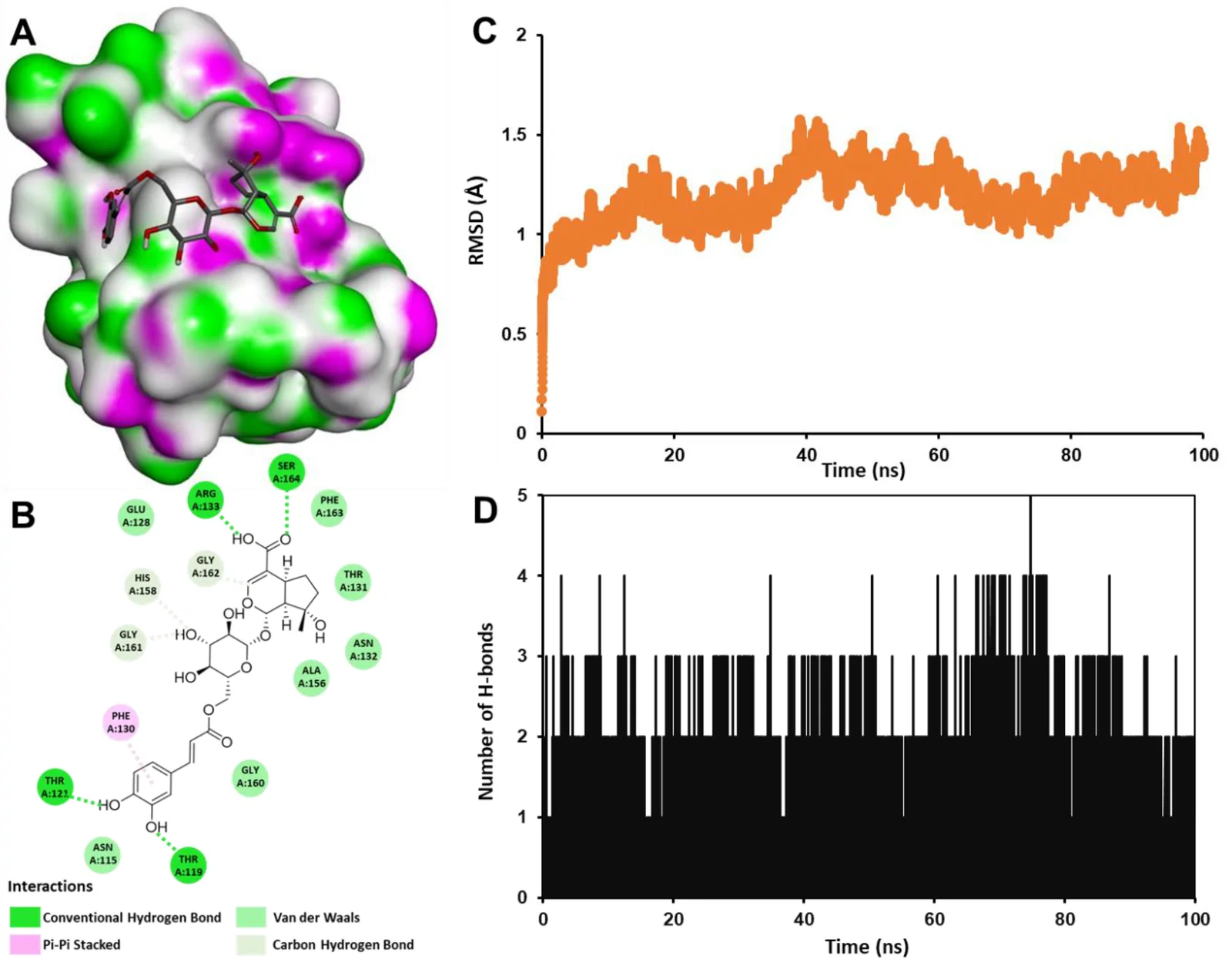
3D molecular docking and 2D interaction diagram of 1QNU protein with compound 8 (A, B), backbone Cα RMSD of the Stx1 B-subunit (C) and the number of intermolecular hydrogen bonds between Stx1 and compound 8 (D) over 100 ns.

Although the flavonoid glycosides isovitexin (compound 7) and luteolin-4′-*O*-glucoside (compound 9) showed ΔG values only 0.2 kcal/mol less favorable than compound 8, we chose the iridoid glycoside for downstream molecular dynamics simulation rather than these phenolic candidates. Polyphenols (including flavonoids, phenolic acids, and their glycosides) have been the subject of extensive experimental and computational investigation regarding Shiga toxin inhibition. Yuan et al. [40] demonstrated that luteolin directly suppresses Stx2-mediated cytotoxicity in Vero cells by disrupting ribosome binding and detailed the underlying hydrogen bonding network at the A-subunit active site. Sheng et al. showed that grape seed extract, rich in oligomeric proanthocyanidins, attenuates Stx production and quorum sensing in non-O157 STEC at sub-inhibitory concentrations [41]. Pellarin et al. similarly documented inhibition of Stx cytotoxicity by polyphenol-rich plant extracts [42]. Taken together, the capacity of flavonoids and related phenolics to interfere with Shiga toxin function is well documented, and reporting yet another flavonoid docking result would add little mechanistic novelty. In contrast, iridoid glycosides, which are a phytochemical hallmark of *V. negundo* (represented here by the chemotaxonomic marker agnuside, compound 5, and the structurally related ester compounds 3, 8, 12 and 17), have not been explored in the context of Stx inhibition to date. Compound 8 matched the reference oxadiazole's affinity while representing a chemically distinct entity; pursuing its dynamics therefore offers a new opportunity for the phytochemical modulation of Stx1 activity, a rationale consistent with the recommendations to prioritize mechanistic novelty over confirmatory findings.

The docking results for Stx2 (PDB ID: 3MXG) presented a different phenomenon (Table S2). While the Oxa control obtained strong affinity for this target (ΔG = −7.0 kcal/mol, K*i* = 7.3 µM), the *V. negundo* metabolites, as a group, exhibited consistently weaker binding energies, predominantly in the range of −4.2 to −6.7 kcal/mol. Even the best-performing putatively annotated compounds - compound 20 (ΔG = −6.7 kcal/mol), compound 9 (ΔG = −6.5 kcal/mol), compounds 4 and 43 (both at ΔG = −6.4 kcal/mol) - fell perceptibly short of the reference oxadiazole's score. Compound 8, which topped the Stx1 rankings, achieved only ΔG = −6.1 kcal/mol (K*i* = 33.6 µM) against Stx2, representing a roughly four-fold reduction in estimated potency. Weak binders such as compounds 50 and 51 (ΔG = −3.8 kcal/mol), and 56 (ΔG = −3.3 kcal/mol) further underscore the limited overall compatibility between the *V. negundo* metabolome and the Stx2 binding pocket. This systematic downward shift in affinity is unlikely to reflect a computational artifact, given that the Oxa control performed comparably against both toxins; instead, it points to structural differences in the active site of Stx1 and Stx2 that favor binding of the plant secondary metabolites to Stx1. The two Shiga toxin variants share approximately 56% amino acid identity in the B-subunit, and structural studies have established that the galactose-binding grooves of Stx1 and Stx2, while topologically similar, differ in key peripheral residues that govern ligand selectivity [23]. The broader and more electropositive character of the Stx1 pocket appears better suited to accommodate the polyhydroxylated, large-surface-area scaffolds characteristic of iridoid and flavonoid glycosides.

### 3.4 Molecular dynamics simulation of the Stx1–compound 8 complex

The Stx1−compound 8 complex was simulated for 100 ns, and the conformational stability of the protein was monitored as the RMSD of the backbone Cα atoms relative to the minimized starting structure (Fig. 5C). During the first ∼10 ns, the system underwent a characteristic equilibration transient in which the RMSD rose sharply from 0 to approximately 1.4 − 1.5 Å, reflecting the structural relaxation of the protein–ligand interface as the solvent shell reorganized around the complex under NPT ensemble conditions. After this initial adjustment, the trajectory plateaued. It remained essentially flat between 0.8 and 1.5 Å for the remainder of the simulation. The mean RMSD across the production phase (10−100 ns) was below 1.5 Å, a threshold widely used in the computational chemistry literature to indicate a well-equilibrated protein structure [43]. The Stx1 B-subunit backbone therefore retained its folded conformation throughout the 100 ns trajectory in the presence of compound 8 under physiologically relevant conditions (310 K, 0.15 M NaCl, TIP3P solvent), with no indication of partial unfolding. Backbone RMSD reports protein conformational stability rather than ligand-binding stability; ligand RMSD, ligand RMSF and binding free energies were not computed in the present study. The hydrogen-bond analysis presented below is therefore the only ligand-centric evidence available here.

The time dependence of intermolecular hydrogen bonds between compound 8 and the Stx1 B-subunit (Fig. 5D) provides the nature of this interaction. Throughout the 100 ns trajectory, the instantaneous H-bond count fluctuated predominantly between 1 and 4, with a modal value of 2 − 3 bonds at any given snapshot. Brief episodes of four or five simultaneous H-bonds were observed at scattered intervals, particularly between approximately 20−30 ns and 70−80 ns. Conversely, transient drops to a single hydrogen bond were also recorded but were short-lived, recovering to the 2 − 3 bond baseline within a few nanoseconds. This dynamic profile is characteristic of both the iridoid glucosyl moiety and the catechol-propenoyl appendage of compound 8, which allows continuous reorganization of the H-bond donor–acceptor pairs without complete displacement from the binding site. A persistent ensemble of 2 − 3 hydrogen bonds over the full 100 ns timescale is generally considered consistent with retention of a small molecule within a protein cavity against thermal fluctuations at 310 K [44].

H-bond occupancy analysis (Table 3) reveals that His158 and Asn132 were the two most persistently engaged residues throughout the simulation, with occupancy values of 24.35% and 16.08%, respectively. In both cases, the ligand acted as an H-bond donor. At the same time, the protein residue served as the acceptor, indicating that the polar groups on compound 8 maintained directed contacts toward these side chains for a substantial fraction of the trajectory. His158 sits at the junction of the β4 strand and the adjacent loop of the Stx1 B-subunit, a region that exhibits mild conformational breathing in the unliganded state [23]; the high occupancy observed here suggests that compound 8 restricts this local flexibility. Asn132 (16.08%) lies within the galactose-binding groove and thus occupies the same spatial region as the physiological Gb3 receptor. A second group of contacts at the Thr121 side chain (6.23%), Gly162 main chain (5.78%), and Glu128 side chain (4.86%) also contributed meaningfully to complex stability. Thr121 and Gly162 are conserved residues that coordinate the galactose moiety of Gb3 in the native Stx1−receptor complex, as established by crystallographic data [23]; their recurrence in the MD trajectory therefore suggests that compound 8 partially recapitulates the binding geometry of the natural carbohydrate ligand. All remaining contacts fell below 4% occupancy and are best treated as transient contributions rather than defining features of the interaction. It is worth noting that the dominant MD contacts − His158, Asn132, Thr121, Gly162 − differ somewhat from the residues identified in the static docking pose (Thr119, Thr121, Arg133, Ser164). This shift is not unusual: docking captures a single low-energy geometry, whereas MD samples the full range of thermally accessible configurations, so the simulation-derived occupancies should be considered more important for understanding how compound 8 behaves at the binding site over time [45].

**Table 3:** Percentage of H-bond occupancy between the Stx1 protein (PDB ID: 1QNU) and compound 8.

No.	H-bond donors	H-bond acceptors	% H-bond occupancy
1	LIGAND-Side	THR119-Side	0.08%
2	ASN115-Side	LIGAND-Side	0.13%
3	LIGAND-Side	PHE163-Main	0.20%
4	LYS113-Side	LIGAND-Side	0.22%
5	ASN132-Side	LIGAND-Side	0.33%
6	LIGAND-Side	SER164-Side	0.40%
7	LIGAND-Side	ASN115-Side	0.89%
8	ARG133-Side	LIGAND-Side	1.52%
9	LIGAND-Side	THR121-Side	1.87%
10	ASN132-Main	LIGAND-Side	3.04%
11	SER164-Side	LIGAND-Side	3.66%
12	LIGAND-Side	GLU128-Side	4.86%
13	GLY162-Main	LIGAND-Side	5.78%
14	THR121-Side	LIGAND-Side	6.23%
15	LIGAND-Side	ASN132-Side	16.08%
16	LIGAND-Side	HIS158-Side	24.35%

### 3.5 ADMET profiling of compound 8

Compound 8 was the top-ranked ligand against Stx1 in molecular docking (ΔG = −6.9 kcal/mol), and the hydrogen-bond profile over the 100 ns MD simulation was consistent with retention of compound 8 in the galactose-binding groove, during which the protein backbone remained conformationally stable (Cα RMSD < 1.5 Å) and recurrent hydrogen bonds were observed at His158 and Asn132. These results provided the rationale for evaluating its predicted ADMET profile. Compound 8 has a LogP of -0.4196, a TPSA of 212.67 Å^2^, seven hydrogen-bond donors, and thirteen hydrogen-bond acceptors, exceeding the limits set by Lipinski's Rule of Five and Veber's criteria and predicting poor passive intestinal permeation [30]. Accordingly, human intestinal absorption was estimated at 9.305%, and the compound falls outside the white region of the BOILED-Egg model [46]. Compound 8 is also predicted to be a P-glycoprotein substrate, which would further reduce systemic uptake by driving active efflux back into the gut lumen [31], consistent with the low bioavailability score of 0.11 [30]. Aqueous solubility (log mol/L = −2.926) is moderate, sufficient for dissolution in gastrointestinal fluids. A predicted logBB of −1.726 indicates that the compound does not cross the blood-brain barrier [31]. No CYP450 inhibition was predicted, and total clearance was low (0.563 log mL/min/kg), suggesting limited hepatic metabolism of any absorbed fraction and extending the potential duration of luminal exposure [31]. On the toxicity side, compound 8 was predicted to be non-mutagenic (AMES negative), to show no hERG I/II inhibition, and to have an oral acute LD50 of 3.241 mol/kg in rats, with a chronic toxicity value of 4.235 log mg/kg_bw/day. These are computational predictions, not a safety assessment; experimental toxicology would be required before considering any oral administration (Table 4).

**Table 4:** ADMET-predicted values for compound 8 using the SwissADME and pkCSM web services.

No.	ADMET properties	Value	No.	ADMET properties	Value
1	Log P	−0.4196	11	AMES toxicity	No
2	TPSA (Å^2^)⁎	212.67	12	Max tolerated dose [log mg/(kg.d)]	−0.215
3	HB donor⁎	7	13	Cytochrome P450 inhibitors	No
4	HB acceptor⁎	13	14	hERG I inhibitor	No
5	Aqueous solubility (log mol/L)	−2.926	15	hERG II inhibitor	No
6	Human intestinal absorption (%)	9.305	16	Oral rat acute toxicity, LD50 (mol/kg)	3.241
7	Blood-brain barrier permeability (logBB)	−1.726	17	Oral rat chronic toxicity (log mg/kg_bw/day)	4.235
8	P-glycoprotein substrate	Yes	18	Skin sensitization	No
9	Total clearance [log ml/(min.kg)]	0.563	19	*T. pyriformis* toxicity (log µg/L)	0.285
10	Bioavailability score⁎	0.11	20	Minnow toxicity (log mM)	2.423

⁎ : using SwissADME web server; other predicted indicators using pkCSM web server.

The gut-restricted distribution profile of compound 8 offers a potential advantage in STEC O157:H7 infections. When administered orally, this compound could achieve therapeutic concentrations directly within the intestinal environment, where it may interfere with Stx1 binding to Gb3 receptors on intestinal epithelial cells. This mechanism could reduce toxin internalization and, in turn, lower the risk of developing hemolytic uremic syndrome [47]. Importantly, the minimal systemic absorption of compound 8 reduces concerns about generating antibiotic selective pressure, which conventionally leads to enhanced Shiga toxin production through activation of the bacterial SOS response [4,9]. These findings suggest that compound 8 is a promising lead for developing gut-restricted anti-Shiga-toxin agents. This approach represents a novel strategy that avoids the complications associated with systemic antibiotic therapy in STEC infections. However, these predictions are computational and need confirmation by wet-lab experiments, particularly Caco-2 permeability assays and in vitro Stx1-neutralization assays.

### 3.6 Physical, antibacterial, and biodegradable properties of the prepared film

A biodegradable film containing 10% glycerol, 10% extract, and 1.5% citric acid, with a thickness of 0.14 ± 0.02 mm, was tested for tensile strength according to the modified ASTM D882 standard. The measurement indicated a tensile strength of ∼23.28 MPa for this film. For comparison, our group previously tested a film with the same base composition (10% glycerol, 10% extract) but without citric acid, using the same ASTM D882 method, and found a tensile strength of 4.26 MPa [26]. Since citric acid was the only difference between the two formulations, this comparison gives a meaningful indication of its crosslinking effect, even though the two films were not tested at the same time. Batch-to-batch variation arising from differences in starch source, extract composition, or humidity during casting cannot be ruled out and may explain part of the difference. Still, the higher tensile strength found in this study fits with citric acid acting as a crosslinking agent, forming a dense 3D network with starch and making the film mechanically stronger [[48], [49], [50]].

The biodegradable film was then evaluated for antibacterial activity using the standard plate count method. As shown in Fig. 6A, the commercial PP plastic had no inhibitory effect on STEC O157:H7, with bacterial survival remaining comparable to that in the negative control. The starch-based film containing only glycerol displayed a slight reduction, likely due to a physical barrier effect rather than actual biological inhibition. However, when combined with *V. negundo* leaf extract, the bacterial population was significantly reduced, confirming its antimicrobial bioactivity. The most remarkable result was observed in the film containing both the extract and citric acid, which exhibited the strongest antibacterial activity. Citric acid likely acts synergistically by lowering the surface pH and functioning as a cross-linker, thereby enhancing the controlled release of the extract's active compounds [48,51].

**Fig. 6:**
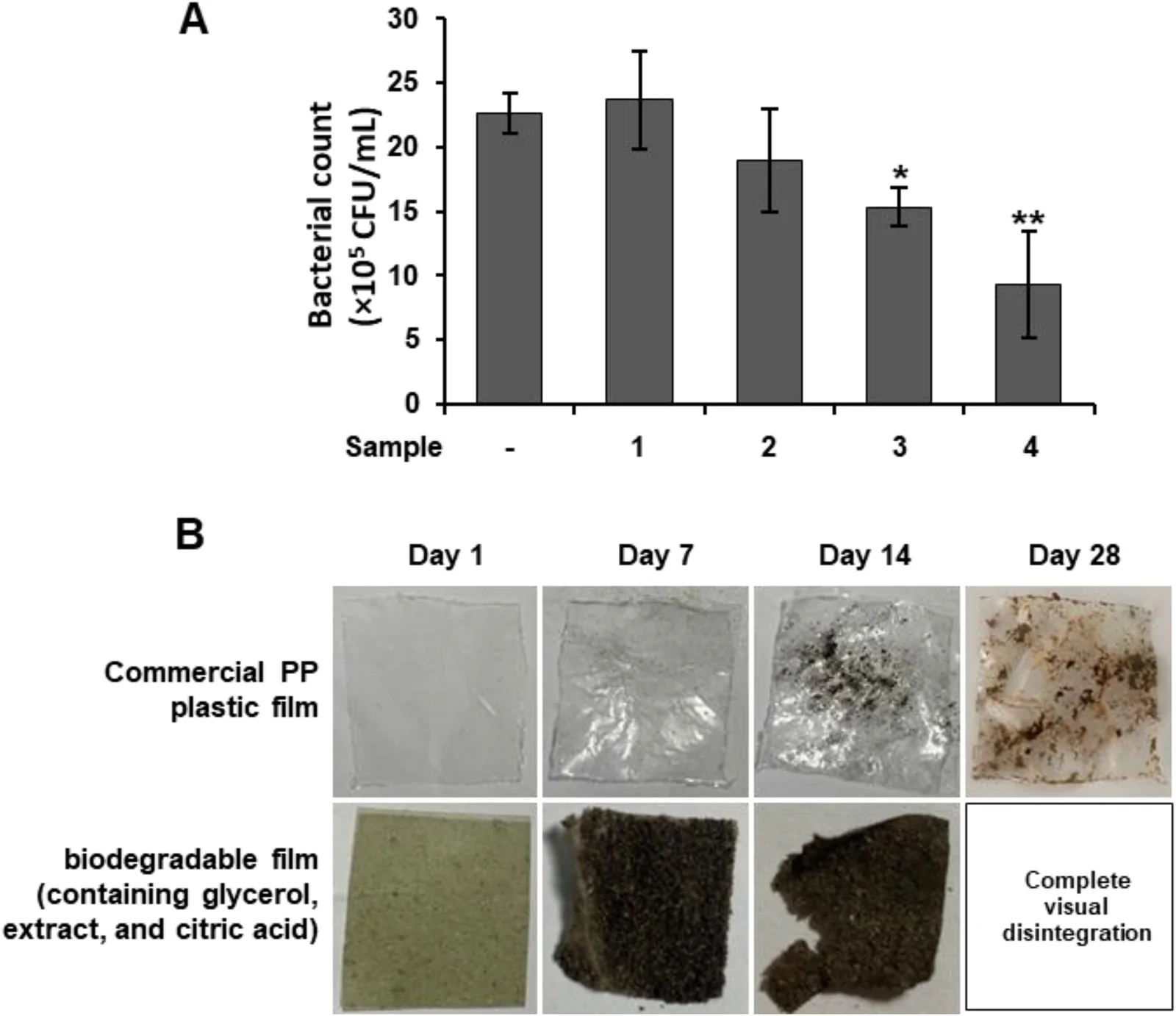
The inhibition activity of plastic films [commercial PP plastic film (1); biodegradable film containing glycerol only (2), glycerol + extract (3), and glycerol + extract + citric acid (4)] against *E. coli* O157:H7 (A). Results are expressed as mean ± SD (*n* = 3 independent experiments); statistical comparisons versus the bacterial (no-film) control were made by one-way ANOVA with Dunnett's test; * *p* < 0.05, ** *p* < 0.01. Degradation properties of commercial PP plastic and biodegradable films during 28 days of soil burial (B).

Regarding environmental effects, Fig. 6B shows the biodegradability of the films during a 28-day soil burial test. The commercial PP film remained completely intact throughout the period, highlighting its resistance to microbial breakdown. Conversely, the prepared film disintegrated rapidly. By day 7, it began to shrink and lose its structural integrity due to moisture absorption. The decomposition accelerated by day 14, with the film breaking into dark, fragile fragments as soil microorganisms actively metabolized the organic starch matrix. By day 28, the bioplastic showed complete visual disintegration, with no macroscopic fragments remaining; this study did not perform gravimetric or chemical confirmation of mineralization, such as respirometry or CO_2_ evolution. Therefore, adding *V. negundo* extract and citric acid enhanced the film's tensile strength and antibacterial activity without impairing its disintegration during soil burial, making it a promising eco-friendly alternative for active packaging of dried foods.

## 4 Conclusion

The application of FBMN-GNPS to the *V. negundo* leaf extract yielded a structurally diverse chemical map of nine structure classes, providing a substantially broader phytochemical foundation than previously available for this species. This metabolite inventory, combined with systematic *in silico* screening, identified compound 8 − an iridoid glycoside − as the candidate with the most promising predicted Stx1-binding potential, distinguished by its equivalent affinity to the synthetic oxadiazole reference, by persistent hydrogen bonding with key residues of the galactose-recognition groove against a conformationally stable protein backbone over 100 ns of molecular dynamics, and by a gut-restricted ADMET profile that circumvents the systemic antibiotic pressure contraindicated in STEC infections. The selective preference of *V. negundo* metabolites for Stx1 over Stx2 is consistent with the structural specificity of the iridoid scaffold toward the Stx1 B-subunit pocket. At the applied level, citric acid crosslinking of starch-based films incorporating the crude extract successfully reconciled mechanical performance, antibacterial efficacy, and complete visual disintegration within 28 days, a combination rarely achieved in conventional packaging systems. Collectively, these findings suggest that *V. negundo* is a dual-purpose botanical resource: a source of iridoid-based antitoxins warranting in vitro validation of Stx1 neutralization, and a functional ingredient for eco-compatible food-safety applications targeting STEC O157:H7.

## Funding

This study is funded by Can Tho University, Code: CTCS2024-02-01.

## CRediT authorship contribution statement

**Nguyen-Huan Pham-Khanh:** Writing – review & editing, Writing – original draft, Supervision, Methodology, Formal analysis, Data curation. **Thi-Kim-Quy Ha:** Writing – review & editing, Visualization, Methodology. **Do-Thanh-Phong Truong:** Methodology.

## Declaration of competing interest

The authors declare that they have no known competing financial interests or personal relationships that could have appeared to influence the work reported in this paper.

## Data Availability

Data will be made available on request.

## References

[1] Xu C., Xin Z., Yu R., Li J., Dan W., Dai J. (2025). The control strategies for *Escherichia coli* O157: H7 in food processing at the physical, chemical and biological levels. Front. Microbiol..

[2] Liu Y., Thaker H., Wang C., Xu Z., Dong M. (2022). Diagnosis and treatment for Shiga toxin-producing *Escherichia coli* associated hemolytic uremic syndrome. Toxins (Basel).

[3] Torres A.G., Amaral M.M., Bentancor L., Galli L., Goldstein J., Krüger A., Rojas-Lopez M. (2018). Recent advances in Shiga toxin-producing *Escherichia coli* research in Latin America. Microorganisms.

[4] Mühlen S., Dersch P. (2020). Treatment strategies for infections with Shiga toxin-producing *Escherichia coli*. Front. Cell. Infect. Microbiol..

[5] Puligundla P., Lim S. (2022). Biocontrol approaches against *Escherichia coli* O157: H7 in foods. Foods.

[6] Duda-Chodak A., Tarko T., Petka-Poniatowska K. (2023). Antimicrobial compounds in food packaging. Int. J. Mol. Sci..

[7] Mody R.K., Griffin P.M. (2016). Editorial commentary: increasing evidence that certain antibiotics should be avoided for Shiga toxin–producing *Escherichia coli* infections: more data needed. Clin. Infect. Dis..

[8] Sheng L., Rasco B., Zhu M.-J. (2016). Cinnamon oil inhibits Shiga toxin type 2 phage induction and Shiga toxin type 2 production in *Escherichia coli* O157: H7. Appl. Environ. Microbiol..

[9] Strzelecki P., Karczewska M., Szalewska-Pałasz A., Nowicki D. (2025). Phytochemicals controlling enterohemorrhagic *Escherichia coli* (EHEC) virulence—current knowledge of their mechanisms of action. Int. J. Mol. Sci..

[10] Munekata P.E.S., Pateiro M., Rodríguez-Lázaro D., Domínguez R., Zhong J., Lorenzo J.M. (2020). The role of essential oils against pathogenic *Escherichia coli* in food products. Microorganisms.

[11] Kotia N., Verma R., Sinha R., Vitex negundo L. (2026). A comprehensive exploration of its phytochemistry, pharmacological attributes and molecular docking insights. Pharmacol. Res. Chinese Med..

[12] Nyamweya B., Rukshala D., Fernando N., de Silva R., Premawansa S., Handunnetti S. (2023). Cardioprotective effects of Vitex negundo: a review of bioactive extracts and compounds. J. Evidence-Based Integr. Med..

[13] Kamal N., Mio Asni N.S., Rozlan I.N.A., Mohd Azmi M.A.H., Mazlan N.W., Mediani A., Baharum S.N., Latip J., Assaw S., Edrada-Ebel R.A. (2022). Traditional medicinal uses, phytochemistry, biological properties, and health applications of Vitex sp. Plants.

[14] Koirala N., Dhakal C., Munankarmi N.N., Ali S.W., Hameed A., Martins N., Sharifi-Rad J., Imran M., Arif A.M., Hanif M.S. (2020). Vitex negundo Linn.: phytochemical composition, nutritional analysis, and antioxidant and antimicrobial activity. Cell. Mol. Biol..

[15] Shettar P.S. (2026). Chemical characterization and In vitro bioactivities of Vitex negundo Leaf extracts. Biomed. Chromatogr..

[16] Wu Q., Zheng J., Yu Y., Li Z., Li Y., Hu C., Zhou Y., Chen R. (2024). Analysis of antioxidant compounds in vitex Negundo leaves using offline 2D-LC-ECD and LC-MS/MS. Molecules.

[17] Wang M., Carver J.J., Phelan V.V, Sanchez L.M., Garg N., Peng Y., Nguyen D.D., Watrous J., Kapono C.A., Luzzatto-Knaan T. (2016). Sharing and community curation of mass spectrometry data with Global Natural Products Social Molecular Networking. Nat. Biotechnol..

[18] Aron A.T., Gentry E.C., McPhail K.L., Nothias L.-F., Nothias-Esposito M., Bouslimani A., Petras D., Gauglitz J.M., Sikora N., Vargas F. (2020). Reproducible molecular networking of untargeted mass spectrometry data using GNPS. Nat. Protoc..

[19] Nothias L.-F., Petras D., Schmid R., Dührkop K., Rainer J., Sarvepalli A., Protsyuk I., Ernst M., Tsugawa H., Fleischauer M. (2020). Feature-based molecular networking in the GNPS analysis environment. Nat. Methods.

[20] Petras D., Phelan V.V, Acharya D., Allen A.E., Aron A.T., Bandeira N., Bowen B.P., Belle-Oudry D., Boecker S., Cummings D.A. (2022). GNPS dashboard: collaborative exploration of mass spectrometry data in the web browser. Nat. Methods.

[21] Trott O., Olson A.J. (2010). AutoDock Vina: improving the speed and accuracy of docking with a new scoring function, efficient optimization, and multithreading. J. Comput. Chem..

[22] SarveAhrabi Y., Khoei S.N. (2024). In vitro and In silico evaluation of antibacterial effects in some oxadiazole compounds against *Escherichia coli* O157: H7. J. Kermanshah Univ. Med. Sci..

[23] Kitov P.I., Sadowska J.M., Mulvey G., Armstrong G.D., Ling H., Pannu N.S., Read R.J., Bundle D.R. (2000). Shiga-like toxins are neutralized by tailored multivalent carbohydrate ligands. Nature.

[24] Phillips J.C., Braun R., Wang W., Gumbart J., Tajkhorshid E., Villa E., Chipot C., Skeel R.D., Kale L., Schulten K. (2005). Scalable molecular dynamics with NAMD. J. Comput. Chem..

[25] Humphrey W., Dalke A., Schulten K. (1996). VMD: visual molecular dynamics. J. Mol. Graph..

[26] Pham-Khanh N.-H., Le H.N.B., Nguyen T.K.T., Ha T.K.Q. (2025). Synthesis of bioplastic films from Vitex negundo L. leaf extract based on sweet potato starch and investigation of antibacterial activity of the products. Quy Nhon Univ. J. Sci..

[27] Elshikh M., Ahmed S., Funston S., Dunlop P., McGaw M., Marchant R., Banat I.M. (2016). Resazurin-based 96-well plate microdilution method for the determination of minimum inhibitory concentration of biosurfactants. Biotechnol. Lett..

[28] Thomas P., Sekhar A.C., Upreti R., Mujawar M.M., Pasha S.S. (2015). Optimization of single plate-serial dilution spotting (SP-SDS) with sample anchoring as an assured method for bacterial and yeast cfu enumeration and single colony isolation from diverse samples. Biotechnol. Reports.

[29] Ahad N.A., Rosli N.A. (2024). Absorption ability and degradable of thin film from orange peel waste. Int. J. Nanoelectron. Mater..

[30] Daina A., Michielin O., Zoete V. (2017). SwissADME: a free web tool to evaluate pharmacokinetics, drug-likeness and medicinal chemistry friendliness of small molecules. Sci. Rep..

[31] Pires D.E.V, Blundell T.L., Ascher pkCSM D.B. (2015). Predicting small-molecule pharmacokinetic and toxicity properties using graph-based signatures. J. Med. Chem..

[32] Echeverría J., Opazo J., Mendoza L., Urzúa A., Wilkens M. (2017). Structure-activity and lipophilicity relationships of selected antibacterial natural flavones and flavanones of Chilean flora. Molecules.

[33] Bello M.O., Zaki A.A., Aloko S., Fasinu P.S., Bello E.O., Ajao U.L., Oguntoye O.S. (2018). The genus Vitex: an overview of iridoids as chemotaxonomic marker, Beni-Suef Univ. J. Basic Appl. Sci..

[34] Feussner I., Wasternack C. (2002). The lipoxygenase pathway. Annu. Rev. Plant Biol..

[35] Boudière L., Michaud M., Petroutsos D., Rébeillé F., Falconet D., Bastien O., Roy S., Finazzi G., Rolland N., Jouhet J. (2014). Glycerolipids in photosynthesis: composition, synthesis and trafficking. Biochim. Biophys. Acta (BBA)-Bioenergetics.

[36] Desbois A.P., Smith V.J. (2010). Antibacterial free fatty acids: activities, mechanisms of action and biotechnological potential. Appl. Microbiol. Biotechnol..

[37] Panda S.K., Thatoi H.N., Dutta S.K. (2009). Antibacterial activity and phytochemical screening of leaf and bark extracts of Vitex negundo l. from similipal biosphere reserve, Orissa. J. Med. Plant Res..

[38] Kamruzzaman M., Bari S.M.N., Faruque S.M. (2013). In vitro and in vivo bactericidal activity of Vitex negundo leaf extract against diverse multidrug resistant enteric bacterial pathogens. Asian Pac. J. Trop. Med..

[39] Morris G.M., Goodsell D.S., Halliday R.S., Huey R., Hart W.E., Belew R.K., Olson A.J. (1998). Automated docking using a lamarckian genetic algorithm and an empirical binding free energy function. J. Comput. Chem..

[40] Yuan L., Nakamichi R., Hirata Y., Matsuda A., Shinohara Y., Yamada A., Masuda Y., Honjoh K., Miyamoto T. (2023). Mechanism for inhibition of cytotoxicity of Shiga toxin by luteolin. Toxicol. Vitr..

[41] Sheng L., Olsen S.A., Hu J., Yue W., Means W.J., Zhu M.J. (2016). Inhibitory effects of grape seed extract on growth, quorum sensing, and virulence factors of CDC “top-six” non-O157 Shiga toxin producing *Escherichia coli*. Int. J. Food Microbiol..

[42] Pellarin M.G., Albrecht C., Rojas M.J., Aguilar J.J., Konigheim B.S., Paraje M.G., Albesa I., Eraso A.J. (2013). Inhibition of cytotoxicity of Shiga toxin of *Escherichia coli* O157: H7 on vero cells by Prosopis alba Griseb (Fabaceae) and Ziziphus mistol Griseb (Rhamnaceae) extracts. J. Food Prot..

[43] Pagadala N.S., Syed K., Tuszynski J. (2017). Software for molecular docking: a review. Biophys. Rev..

[44] Durrant J.D., McCammon J.A. (2011). Molecular dynamics simulations and drug discovery. BMC Biol.

[45] De Vivo M., Masetti M., Bottegoni G., Cavalli A. (2016). Role of molecular dynamics and related methods in drug discovery. J. Med. Chem..

[46] Daina A., Zoete V. (2016). A boiled-egg to predict gastrointestinal absorption and brain penetration of small molecules. ChemMedChem.

[47] Calanni F., Renzulli C., Barbanti M., Viscomi G.C. (2014). Rifaximin: beyond the traditional antibiotic activity. J. Antibiot. (Tokyo)..

[48] Reddy N., Yang Y. (2010). Citric acid cross-linking of starch films. Food Chem..

[49] Seligra P.G., Jaramillo C.M., Famá L., Goyanes S. (2016). Biodegradable and non-retrogradable eco-films based on starch–glycerol with citric acid as crosslinking agent. Carbohydr. Polym..

[50] Ghanbarzadeh B., Almasi H., Entezami A.A. (2011). Improving the barrier and mechanical properties of corn starch-based edible films: effect of citric acid and carboxymethyl cellulose. Ind. Crops Prod..

[51] Burt S. (2004). Essential oils: their antibacterial properties and potential applications in foods—a review. Int. J. Food Microbiol..

