# Supplementary material for: UPLC-MS/MS-GNPS based metabolite profiling and inhibitory potential of Vitex negundo L. leaves against Shiga toxins: Applications for biodegradable films with antibacterial properties
Source: Biotechnol Rep (Amst). 2026 Oct 19;52:e00987. doi: 10.1016/j.btre.2026.e00987 (PMC13717098; doi:10.1016/j.btre.2026.e00987)
Supplement: Supplementary file 1 [file mmc1.docx]

**SUPPORTING DATA**

UPLC-MS/MS-GNPS based metabolite profiling and inhibitory potential of *Vitex negundo* L. leaves against Shiga toxins: Applications for biodegradable films with antibacterial properties

**Nguyen-Huan Pham-Khanh^a,*^**, **Thi-Kim-Quy Ha^a^, Do-Thanh-Phong Truong^a^**

*^a^ College of Natural Sciences, Can Tho University, Campus II, 3/2 street, Ninh Kieu ward, Can Tho City 94000, Vietnam*

*** Corresponding author:** [**pknhuan@ctu.edu.vn**](mailto:pknhuan@ctu.edu.vn)**.**

**Contents**

Table S1: Molecular docking simulations of Stx1 protein with predicted compounds	2

Table S2: Molecular docking simulations of Stx2 protein with predicted compounds	18

Figure S1: Feature-based Molecular networking of *V. negundo* leaf extract in negative mode.	19

Figure S2: Feature-based Molecular networking of *V. negundo* leaf extract in positive mode.	20

Table S1: Molecular docking simulations of Stx1 protein with predicted compounds

| **No.** | **Bingding**  **energy** | **K*i* value (µM)** | **H-bond**  **number** | **2D interaction** |
| --- | --- | --- | --- | --- |
| 1 | -4.8 | 301.6 | ASN115  THR119  THR121 |  |
| 2 | -4.8 | 301.6 | ASP117  THR119  THR121 |  |
| 3 | -6.4 | 20.2 | ASN132  THR154  ASN155 |  |
| 4 | -6.4 | 20.2 | ARG133  LYS153  THR154  GLU165 |  |
| 5 | -5.9 | 47.1 | THR154  SER164  GLU165 |  |
| 6 | -4.8 | 301.6 | - |  |
| 7 | -6.7 | 12.2 | ARG133  LYS153  THR154  SER164  GLU165 |  |
| 8 | -6.9 | 8.7 | THR119  THR121  ARG133  SER164 |  |
| 9 | -6.7 | 12.2 | ASP117  THR154  SER164 |  |
| 10 | -5.7 | 66.0 | - |  |
| 11 | -4.8 | 301.6 | - |  |
| 12 | -5.7 | 66.0 | ASN132  ASN155  PHE163 |  |
| 13 | -6.6 | 14.4 | ASN132  THR154  SER164 |  |
| 14 | -6.6 | 14.4 | - |  |
| 15 | -5.4 | 109.5 | ASN115  THR121 |  |
| 16 | -4.7 | 357.1 | THR119 |  |
| 17 | -5.3 | 129.6 | THR101  LYS153  THR154  SER164 |  |
| 18 | -5.8 | 55.7 | ASN132  ARG133  SER164 |  |
| 19 | -5.7 | 66.0 | - |  |
| 20 | -6.6 | 14.4 | THR101  ARG133  THR154  SER164  GLU165 |  |
| 21 | -5.5 | 92.5 | SER164 |  |
| 22 | -5.5 | 92.5 | - |  |
| 23 | -5.8 | 55.7 | THR154  GLY162 |  |
| 24 | -5.7 | 66.0 | ASN132  ARG133  GLY162  GLU165 |  |
| 25 | -6 | 39.7 | SER164  GLU165 |  |
| 26 | -5.6 | 78.1 | - |  |
| 27 | -4.7 | 357.1 | ASN132  ARG133  GLY162 |  |
| 28 | -5.7 | 66.0 | THR154 |  |
| 29 | -5.7 | 66.0 | SER164 |  |
| 30 | -6 | 39.7 | THR101  THR154 |  |
| 31 | -5.6 | 78.1 | PHE163  SER164 |  |
| 32 | -5.8 | 55.7 | ARG133  SER164 |  |
| 33 | -5.3 | 129.6 | PHE163  SER164 |  |
| 34 | -6.4 | 20.2 | LYS153 |  |
| 35 | -5.3 | 129.6 | LYS153  SER164 |  |
| 36 | -5.5 | 92.5 | ASN132 |  |
| 37 | -5.5 | 92.5 | ARG133 |  |
| 38 | -6.1 | 33.6 | THR101  ASN132  ARG133  THR154  PHE163 |  |
| 39 | -6 | 39.7 | LYS153 |  |
| 40 | -5.3 | 129.6 | ASN132  ARG133 |  |
| 41 | -4 | 1164.7 | ARG133  LYS153  SER164 |  |
| 42 | -6 | 39.7 | THR101  THR154  PHE163  SER164 |  |
| 43 | -6.6 | 14.4 | THR101  GLY162  GLU165 |  |
| 44 | -4.5 | 500.6 | - |  |
| 45 | -5.4 | 109.5 | LYS153 |  |
| 46 | -5.5 | 92.5 | THR154  ASN155 |  |
| 47 | -5.4 | 109.5 | ASN132  ARG133  PHE163 |  |
| 48 | -4.6 | 422.8 | - |  |
| 49 | -5.7 | 66.0 | - |  |
| 50 | -4.6 | 422.8 | GLU165 |  |
| 51 | -4.7 | 357.1 | ASN132 |  |
| 52 | -4.5 | 500.6 | LYS153 |  |
| 53 | -5.3 | 129.6 | LYS153  SER164 |  |
| 54 | -4.5 | 500.6 | LYS153 |  |
| 55 | -5 | 215.2 | GLY162 |  |
| 56 | -3.7 | 1933.0 | LYS153 |  |
| Oxa | -6.9 | 8.7 | LYS153  THR154  SER164 |  |

Table S2: Molecular docking simulations of Stx2 protein with predicted compounds

| **No.** | **Bingding**  **energy** | **K*i* value** | **No.** | **Bingding**  **energy** | **Ki value** | **No.** | **Bingding**  **energy** | **Ki value** |
| --- | --- | --- | --- | --- | --- | --- | --- | --- |
| 1 | -4.4 | 592.7 | 21 | -6 | 39.7 | 41 | -3.9 | 1378.9 |
| 2 | -4.6 | 422.8 | 22 | -5.7 | 66.0 | 42 | -4.3 | 701.7 |
| 3 | -6 | 39.7 | 23 | -6 | 39.7 | 43 | -6.4 | 20.2 |
| 4 | -6.4 | 20.2 | 24 | -5.7 | 66.0 | 44 | -4.3 | 701.7 |
| 5 | -5.9 | 47.1 | 25 | -6 | 39.7 | 45 | -5.1 | 181.7 |
| 6 | -4.7 | 357.1 | 26 | -5.9 | 47.1 | 46 | -5.2 | 153.5 |
| 7 | -6.2 | 28.4 | 27 | -4.3 | 701.7 | 47 | -5.6 | 78.1 |
| 8 | -6.1 | 33.6 | 28 | -5.7 | 66.0 | 48 | -4.4 | 592.7 |
| 9 | -6.5 | 17.1 | 29 | -6 | 39.7 | 49 | -5.9 | 47.1 |
| 10 | -6 | 39.7 | 30 | -5.7 | 66.0 | 50 | -3.8 | 1632.6 |
| 11 | -4.8 | 301.6 | 31 | -5.8 | 55.7 | 51 | -3.8 | 1632.6 |
| 12 | -6 | 39.7 | 32 | -5.8 | 55.7 | 52 | -3.5 | 2709.7 |
| 13 | -5.7 | 66.0 | 33 | -5.5 | 92.5 | 53 | -4.8 | 301.6 |
| 14 | -6.8 | 10.3 | 34 | -5.8 | 55.7 | 54 | -3.5 | 2709.7 |
| 15 | -4.6 | 422.8 | 35 | -4 | 1164.7 | 55 | -5 | 215.2 |
| 16 | -4.8 | 301.6 | 36 | -5.7 | 66.0 | 56 | -3.3 | 3798.5 |
| 17 | -5.8 | 55.7 | 37 | -5.8 | 55.7 | Oxa | -7 | 7.3 |
| 18 | -5.8 | 55.7 | 38 | -4.8 | 301.6 |  |  |  |
| 19 | -5.8 | 55.7 | 39 | -5.5 | 92.5 |  |  |  |
| 20 | -6.7 | 12.2 | 40 | -4.2 | 830.8 |  |  |  |

Figure S1: Feature-based Molecular networking of *V. negundo* leaf extract in negative mode.

Figure S2: Feature-based Molecular networking of *V. negundo* leaf extract in positive mode.
